# A multiscale landscape approach for prioritizing river and stream protection and restoration actions

**DOI:** 10.1002/ecs2.4350

**Published:** 2023-01-19

**Authors:** Luisa Riato, Scott G. Leibowitz, Marc H. Weber, Ryan A. Hill

**Affiliations:** U.S. Environmental Protection Agency, Center for Public Health and Environmental Assessment, Pacific Ecological Systems Division, Corvallis, Oregon, USA

**Keywords:** Benthic Index of Biotic Integrity, catchment, dispersal, ecological condition assessments, landscape information, multiple spatial scales, stream management, StreamCat, watershed

## Abstract

River and stream conservation programs have historically focused on a single spatial scale, for example, a watershed or stream site. Recently, the use of landscape information (e.g., land use and land cover) at multiple spatial scales and over large spatial extents has highlighted the importance of incorporating a landscape perspective into stream protection and restoration activities. Previously, we developed a novel framework that links information about watershed-, catchment-, and reach-scale integrity with stream biological condition using scatterplots and a landscape integrity map. Here we examined an application of this approach for streams in urban and other settings in King County, Washington State, United States, where we related stream macroinvertebrate condition to two indices of landscape integrity, the US Environmental Protection Agency’s (USEPA) nationally available Index of Watershed Integrity (IWI) and Index of Catchment Integrity (ICI). We generated a scatterplot of IWI versus ICI for sample sites, where points represented site macroinvertebrate condition from poor to good. The same data were also visualized as a landscape integrity map that displayed catchments of King County according to the level of watershed and catchment integrity (high or low IWI/ICI). Almost three-quarters of poor-condition sites were associated with high-integrity watersheds and catchments (i.e., underperforming sites), which suggested that either one or both national indicators were insufficient for this area, and that sites underperformed because of local-scale factors. In response, we used a catchment-scale indicator related to forest condition (PctForestCat) after examining several GIS-based dispersal indicators from the National Hydrography Dataset and other candidates from the USEPA’s StreamCat dataset. We then compared the results of the scatterplots and maps based on the current and original analyses and found that many of the sites previously classified as underperforming now performed as expected, that is, they were poor-condition sites in poor-condition catchments. This analysis demonstrates how results based on a national dataset can be improved by developing an alternative that represents regionally important stressors. The methods used to develop an effective landscape indicator based on StreamCat datasets, and the utility of the multiscale approach, could provide important tools for prioritizing, optimizing, and communicating stream conservation actions.

## INTRODUCTION

Historically, programs charged with the protection and restoration of rivers and streams have focused on reach- or site-scale processes (Flotemersch et al., 2006; USEPA, 1996), yet, increasingly, landscape information is being used to address local catchment- or watershed-scale influences on aquatic biological condition (Ettinger et al., 2021; Tsang et al., 2014; USEPA, 2012). Landscape factors, both natural (e.g., hydrology, topography, soils) and anthropogenic (e.g., urbanization and agriculture), function over large spatial scales to influence stream biological condition at local scales (Allan, 2004; Carlisle et al., 2009). If a site is in poor biological condition, it could be impaired by factors at the reach scale, local catchment scale (i.e., the land area draining to a stream reach, excluding the upstream catchments), or watershed scale (i.e., the area draining to a stream reach, which consists of the local catchment plus all upstream catchments), or some combination. These multiple scales of influence highlight the importance of incorporating the local catchment and watershed characteristics into management actions (Hill et al., 2016, 2017) that may enable a more integrated assessment of condition and the most feasible and/or likely effective management actions (Palmer et al., 2010). For example, restoration of stream condition in an impaired catchment but relatively intact watershed will be more feasible and more likely effective than similar efforts where both the catchment and watershed are impaired, assuming similar site conditions. Conversely, site-level restoration is less likely to be successful in a watershed with extensive upstream urban and/or agricultural nonpoint source pollution (Bernhardt & Palmer, 2011).

With an increasing trend to include a landscape perspective into conservation actions of freshwater systems, several efforts have developed methods using geospatial data to measure landscape influences at a variety of spatial scales (e.g., Hill et al., 2016; Thornbrugh et al., 2018; Wang et al., 2011). Based on data from these efforts, researchers from the US Environmental Protection Agency (USEPA) developed two related indicators of landscape condition, the Index of Watershed Integrity (IWI) and the Index of Catchment Integrity (ICI), to quantify and map the integrity of watersheds and catchments of 2.6 million stream segments across the conterminous United States (CONUS) (Flotemersch et al., 2016; Thornbrugh et al., 2018). Kuhn et al. (2018) evaluated the performance of the IWI and ICI at regional and local watershed scales using ecological response data from four case study watersheds across the United States.

While the IWI and ICI can indicate watershed integrity at multiple spatial scales, only recently has a simple framework been developed that allows the linking of information from three different scales of landscape integrity (stream-reach, catchment, and watershed scales) to biological condition data (Riato et al., 2020). In the study by Riato et al. (2020), scatterplots and a landscape integrity map were used to relate samples of stream condition classes to stream-reach-, catchment-, and watershed-scale integrity. Identifying the quadrant in the scatterplot or catchment in the landscape integrity map in which a good- or poor-condition sample is located may allow for distinct management actions, for example, samples from sites that have high integrity (e.g., high IWI/ICI values) would be good candidates for protection or restoration. These different types of data, visualized in a scatterplot and landscape integrity map, could provide managers with a powerful tool for identifying and prioritizing sites for protection or restoration, a task that has become increasingly challenging in a rapidly changing, human-altered landscape (Ettinger et al., 2021).

Riato et al. (2020) recently related IWI and ICI values taken from EPA’s StreamCat dataset (Hill et al., 2016) to stream macroinvertebrate condition data collected across the Puget Lowland, including King County, developing a multiscale scatterplot and landscape integrity map. We generally expect sites in high-integrity watersheds and catchments to be in good condition. While a few poor-condition sites would be expected, due to the occurrence of poor local conditions, almost three-quarters of poor-condition stream sites (77 of 110 sites) were unexpectedly associated with high-integrity watersheds and catchments (IWI and ICI values >0.5; herein defined as underperforming sites, which is based on the scatterplot and Benthic Index of Biotic Integrity [B-IBI] score combined). This is unexpected because this suggests a trend in poor conditions that would not occur in high-integrity watersheds and catchments (if the poor conditions were due to extensive human impacts, for example, catchment integrity would likely not be high). Thus, we hypothesized that either one or both national indicators were insufficient for explaining degraded biological conditions in this area, and that the sites underperformed because of local-scale factors that are difficult to measure with landscape indicators (e.g., point source impairment). We also hypothesized that sites underperformed because of factors related to dispersal limitation (e.g., lack of in-stream connectivity and availability to high-quality habitat) that may be affecting many streams in King County that are in highly urbanized settings. Urbanization can alter upland and riparian habitat structure and, as such, affect aquatic insect fitness and movement within the stream (Smith et al., 2009).

In this paper, we address this problem by developing an alternative landscape indicator for this area that reduces the number of underperforming sites, and, in so doing, we demonstrate how to replace a default national indicator with a more effective landscape indicator and to develop a tool for better management of streams. Our goals for this study were to: (1) develop a catchment- and/or watershed-scale indicator based on an examination of candidate indicators that includes indicators of dispersal limitation (e.g., measures of in-stream connectivity, habitat quality and quantity), and determine if it better explained macroinvertebrate condition in King County streams; (2) compare the results of the scatterplot and map based on this new indicator with the original scatterplot and map. In this way, we illustrate how the multiscale framework and approach could be modified and applied when prioritizing areas for protection or restoration action. We accomplish these goals by first providing a background of the multiscale landscape framework in the next section.

## METHODS

### Background

**FIGURE 1:**
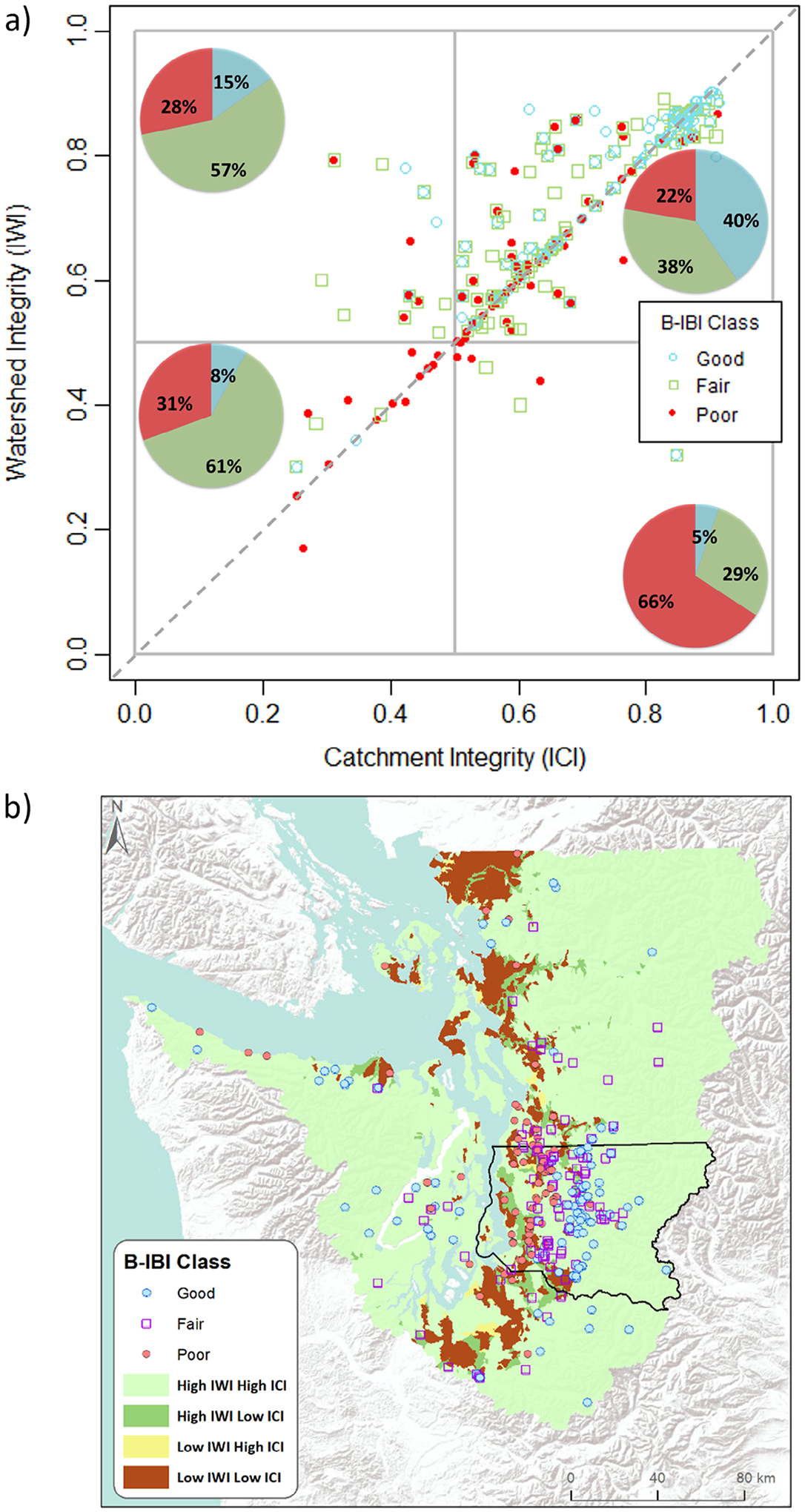
(a) A scatterplot using macroinvertebrate Benthic Index of Biotic Integrity (B-IBI) assessment data for streams and rivers across the Puget Lowland including King County, displaying the relationship between estimates of B-IBI condition (*n* = 782 samples from 280 unique sites, that is a combined dataset collected once per year between July and October from 2009 to 2017 by the King County Water and Land Resources Division (*n* = 583) and by another agency, the Washington Department of Ecology (*n* = 199)) and corresponding Index of Watershed Integrity (IWI) and Index of Catchment Integrity (ICI) values for each sample site from (0) low integrity to (1) high integrity. The different colors of points represent the classes of site condition from poor to good condition. The pie charts display the distribution of each B-IBI class in each quadrant. Dashed line represents the 1:1 relationship between IWI and ICI. (b) A landscape integrity map displaying the catchments of the study area according to the level of watershed and catchment integrity (high or low IWI and ICI), along with locations and most recent conditions of B-IBI sample sites (Riato et al., 2020).

We used scatterplots to investigate the role of multiple scales of landscape integrity on ecological indicators. In a multiscale scatterplot, the *x*- and *y*-axes must represent two different scales of landscape integrity. For example, the *x*-axis and *y*-axis could represent local catchment and watershed integrity, respectively, in which the nationally available ICI and IWI could be used as default indicators, but other indicators are possible (Riato et al., 2020). Four quadrants are generated in the scatterplot by drawing two perpendicular lines (e.g., positioned at the 0.5 values) of each landscape integrity index on a 0–1 scale (Figure 1a). We chose 0.5 to represent the threshold between good and poor conditions because it is a simple and parsimonious approach. Note that managers could define the quadrants using other thresholds. Each stream site has a corresponding catchment and watershed integrity value that is plotted as a point on the scatterplot, where the color of the point represents the condition of the site based on some independent site-based measure. The purpose of the multiscale landscape framework is not to develop a model to determine empirical relationships between landscape integrity indices and biological condition data but to present a simple screening method that (1) shows general relationships that could be useful to stream management and (2) does not rely on complex modeling to facilitate its use and application. A simple approach can be useful and preferred in a management environment where data availability and modeling expertise can be more limiting than in research environments, and where decision-making needs to be accomplished more rapidly.

Riato et al. (2020) demonstrated the practical application of using multiscale scatterplots with macroinvertebrate condition class data as a site-based measure for river and stream locations across the Puget Lowland, including King County, Washington State. A scatterplot of IWI (*y*-axis) versus ICI (*x*-axis) was used for stream sampling sites, where the points represented site condition from poor (red) to good (blue) based on a B-IBI (Figure 1a). The IWI and ICI concepts were proposed by Flotemersch et al. (2016) and applied (Flotemersch et al., 2016; Thornbrugh et al., 2018) by using the framework of the 1:100 K scale National Hydrography Dataset Plus Version 2 (NHDPlusV2; McKay et al., 2012). This included 23 anthropogenic factors taken from the USEPA’s StreamCat dataset (e.g., percent urban or agriculture in watershed; Hill et al., 2016), to map potential high and low watershed and catchment integrity. The StreamCat dataset contains hundreds of watershed metrics of land use, land cover, climate, and other landscape features at the catchment and watershed scales for 2.65 million stream segments across the CONUS that are at the 1:100 K scale, which may be inadequate at a local level, for example, because it would not include finer streams that would be important at a local level. The IWI and ICI were developed such that as stressors increase within a watershed or catchment, the integrity declines (Flotemersch et al., 2016). Both the IWI and ICI have values that vary from 0 to 1, where 1 represents high integrity. The IWI and ICI can also be represented as stressors on a 1–0 scale, that is, 1 – the integrity value.

The same datasets were also visualized as a landscape integrity map that displayed the catchments of the Puget Lowland/King County area according to the level of watershed and catchment integrity (high or low IWI and ICI), along with the locations and conditions of the sample sites (Figure 1b). Identifying the most appropriate sites for protection or restoration could be inferred using the IWI/ICI scatterplot or landscape integrity map. Table 1 summarizes the different management applications of the quadrants in the scatterplot and catchment classes in the landscape integrity map.

Sites in the upper right quadrant in the scatterplot, or catchment in the landscape integrity map (i.e., high IWI and ICI values, Figure 1a, and light green catchments, Figure 1b), where a good- or poor-condition site (i.e., underperforming site) is located, could be high-priority sites for site-level protection or restoration since local efforts would likely be feasible and effective if the watershed and catchment integrity are good. Protection or restoration activities are more likely to be successful in relatively intact watersheds and catchments because the stressors are likely simpler and come from fewer sources. There may be exceptions, however, to the feasibility and effectiveness of such activities in healthy landscapes. For example, if a site with an intact watershed/catchment is below a dam where removal is highly infeasible, then protection or restoration activities are less likely to succeed. Similarly, sites in good or poor condition that have high watershed integrity but lower catchment integrity (i.e., high IWI but low ICI values in the upper left quadrant, Figure 1a; and dark green catchments, Figure 1b) could provide further opportunities for effective and efficient management since a healthy watershed can support protection and restoration actions responding to stressors at the catchment level, as hypothesized in Hill et al. (2017). In contrast, remediation efforts within watersheds with low integrity (i.e., low IWI values in the lower two quadrants, Figure 1a; and yellow and brown catchments, Figure 1b) are unlikely to sustain restoration without responding to upstream (watershed scale) stressors that could require considerable effort and resources (Aho et al., 2020). We note, however, that these classifications are not an endpoint, but are a screening approach to identify a pool of candidate sites that management could follow up on at an individual site and further refine the list of candidate streams. Some misalignment can occur due to unaccounted-for localized factors. In such cases, good-condition sites can be unexpectedly in the lower two quadrants (represented as yellow and brown catchments), within low-integrity catchments and/or watersheds (herein, defined as *overperforming* sites), or poor-condition sites are in the upper right quadrant (represented as light green catchments), within high-integrity catchments and watersheds (i.e., underperforming sites), as was the case with most poor-condition stream sites across the Puget Lowland including King County (Figure 1a,b). This larger than expected number of underperforming sites could be due to two factors: (1) the IWI or ICI (or both) is not properly functioning as an indicator of catchment- or watershed-scale integrity, possibly because the ICI and IWI are national indicators and so are not sensitive to regionally important factors; and/or (2) there are factors at other scales (e.g., the local reach scale) that are causing the underperformance.

We evaluated the potential factors that could explain the large number of underperforming sites that were likely relevant to King County. Specifically, (1) factors that affect watershed/catchment integrity in this area that the national IWI/ICI were unable to reflect, and (2) factors related to dispersal limitation (e.g., proximity to high-quality habitat). Monitoring activities in King County suggest that the poor biological condition at underperforming sites is due to a combination of watershed-, catchment-, and more local-scale factors such as extent of urban development, excessive fine sediments, and poor water quality in most degraded watersheds. In geographically isolated watersheds, poor B-IBI scores may also be a result of regional-scale factors limiting the dispersal of macroinvertebrates to these streams (Clinton et al., 2022; King County, 2020b). The idea that dispersal limitation could be a contributing factor explaining the poor biological condition is based on the fact that many streams in King County are in highly urbanized locations, where fragmented stream systems can restrict or prevent movement of stream macroinvertebrates (Crook et al., 2015; McKinney, 2002). Also, in most urban areas of King County, it is common to have small streams much farther than 5 km from any high-quality streams, and this distance could limit the availability of healthy source populations for those streams (Clinton et al., 2022; King County, 2020b). In the sections below, we describe how we developed a landscape indicator based on an examination of dispersal indicators and other suitable candidates to determine if that would result in fewer underperforming sites.

### Study area

**FIGURE 2:**
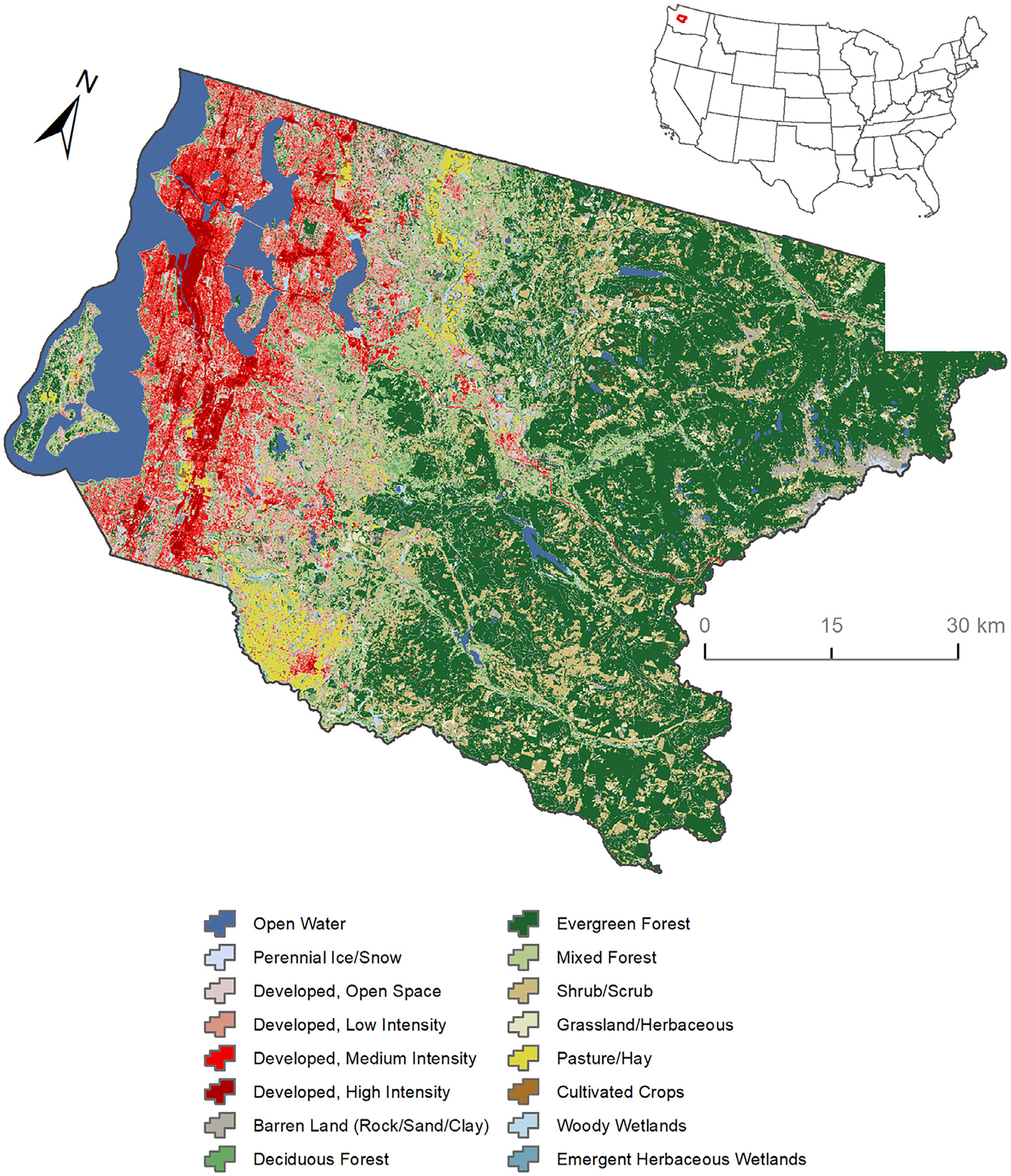
Map of the King County study area showing land use and land cover features using 2016 National Land Cover Data. Inset: map showing location of King County within Washington State (WA) and the conterminous United States.

King County covers approximately 6000 km^2^ and is the most populated county in Washington State. Western King County is dominated by urban development (Seattle metropolitan area with a population of approximately 2.2 million) located along the Puget Sound, the second largest estuary in the United States. The east is predominately forested, and agriculture comprises most of the north and south (Sheibley et al., 2017; Figure 2). King County is located within the Puget Lowland ecoregion (Omernik & Griffith, 2014), which encompasses almost 14,000 km^2^. The Puget Lowland has a mild, maritime climate (USEPA, 2013) with an average summer temperature of 15°C and average winter temperature of 3.5°C. Almost 75% of the region’s annual precipitation occurs between October and March with an average annual precipitation of approximately 1000 mm/year at or near sea level and increasing with elevation. Streams in King County range in elevation from sea level to 2400 m, where rainfall is the primary source of streamflow, with highest flows between November and March, and lowest flows between July and September (DeGasperi et al., 2009). The study area encompasses 15,210 km of streams that are characterized by low-gradient, pool-riffle habitats (May et al., 1997).

### Analytical approach

Multiple linear regression is commonly used to assess the response of biological communities to several environmental factors (explanatory or predictor variables; James & McCulloch, 1990). Such an analysis allows the strength of the relationship between a response variable and predictor variables to be assessed, as well as the importance of each of the predictors to the relationship, with the effect of other predictors statistically removed (Graham, 2003). We used aquatic macroinvertebrate stream condition data derived from the Puget Lowland B-IBI as the response variable for the multiple regression. The B-IBI is a long-established and widely used (including by King County, 2014) macroinvertebrate multimetric index (Karr, 1998; Morley & Karr, 2002) to evaluate biological degradation and restoration efforts in streams throughout the Puget Lowland. The data (*n* = 938 samples) were collected from 177 wadeable stream sites once per year between July and October from 2012 to 2019 by the King County Water and Land Resources Division as part of their Freshwater Benthic Macroinvertebrate Monitoring Program following standard protocols (King County, 2020a). Data and details of the assessment are available at: https://pugetsoundstreambenthos.org/; and in King County (2020a). Macroinvertebrate assemblage data used to produce the B-IBI scores were based on samples with at least 300 counts with a target count of 500. We combined B-IBI scores for every sample collected at a site during those years, to produce a set of mean B-IBI scores (*n* = 177) as the response variable for the multiple regression.

### Multiple regression—ensuring and assessing model quality

The predictor variables differed in units, making comparison of parameters among predictors difficult. We therefore centered and scaled all predictors to have a mean of zero and standard deviation of one. To address potential overfitting, we used a backward–forward stepwise regression, based on the Akaike information criterion (AIC) (Cavanaugh, 1997), which is a way of selecting a parsimonious model. Multicollinearity among predictor variables was controlled for by monitoring the variance inflation factors (VIF), a metric that indicates the degree to which the variance of an individual predictor regression coefficient is increased due to collinearity. Variables with a VIF greater than 2.5 were dropped from the analysis. Moreover, models were assessed such that all predictor variables were significant in the model (α = 0.05) and that the models themselves were significant. The final model was assessed in terms of its adherence to the assumptions of multiple regression by inspecting the normality of the distribution of residuals and the homoscedasticity of residuals using the Breusch–Pagan test (Breusch & Pagan, 1979), as well as a visual interpretation of scatterplots of residuals versus predicted values.

### Comparisons between original and revised scatterplots

To determine if the top model predictors at the catchment or watershed scale would result in fewer underperforming sites, we substituted the top catchment predictor variable from the final regression model for our catchment indicator, the ICI, or top watershed predictor variable for our watershed indicator, the IWI, and used this to examine B-IBI classes. Similar to Riato et al. (2020), to obtain B-IBI condition classes, we categorized B-IBI scores into good, fair, and poor biological conditions based on condition class thresholds for B-IBI scores in the Western Washington region where the sample sites were located. B-IBI scores ≤49.98 were classified as poor, scores ≥73.73 were classified as good, and those between 49.98 and 73.73 were considered fair (Larson et al., 2019). Note that other agencies, jurisdictions, and tribes may use a different B-IBI scoring system to categorize scores into good, fair, and poor conditions (King County, 2020a). In a scatterplot, we showed the relationship between B-IBI condition and the original corresponding IWI and ICI values for each sample site; and in a separate scatterplot, the relationship between B-IBI condition and the revised watershed or catchment predictors for each site. Predictor values were transformed to a scale from 0 to 1 (1 being high integrity). For predictors that were based on percentages, like percent forest cover, we would divide by 100 so final values range from 0 to 1. For predictors that were not based on a proportion and had no theoretical limit (e.g., road-stream crossings upstream), we would winsorize (Gilbert, 1987) the data to change extreme values in a dataset (extreme high and low) to less extreme values. We would use a 90% winsorization, which sets all observations greater than the 95th percentile equal to the value at the 95th percentile, and all observations less than the 5th percentile equal to the value at the 5th percentile. This would limit extreme values in the statistical data to minimize the influence of outliers in the data. We would then find the maximum value over all catchments, *c*_max_, and divide each watershed or catchment value by *c*_max_ to get a value that ranges from 0 to 1. Finally, we classified the revised watershed or catchment predictors into high (≥0.5) or low (≤0.5) and mapped the mean B-IBI condition of sites based on the revised watershed and catchment values.

### Predictor variables

We developed a set of predictor variables for inclusion in the multiple regression that represented different factors that have been shown in the literature to influence stream biological condition (Table 2). In the sections below, we provide a description of how we derived those predictors.

### Indicators of dispersal limitation

**FIGURE 3:**
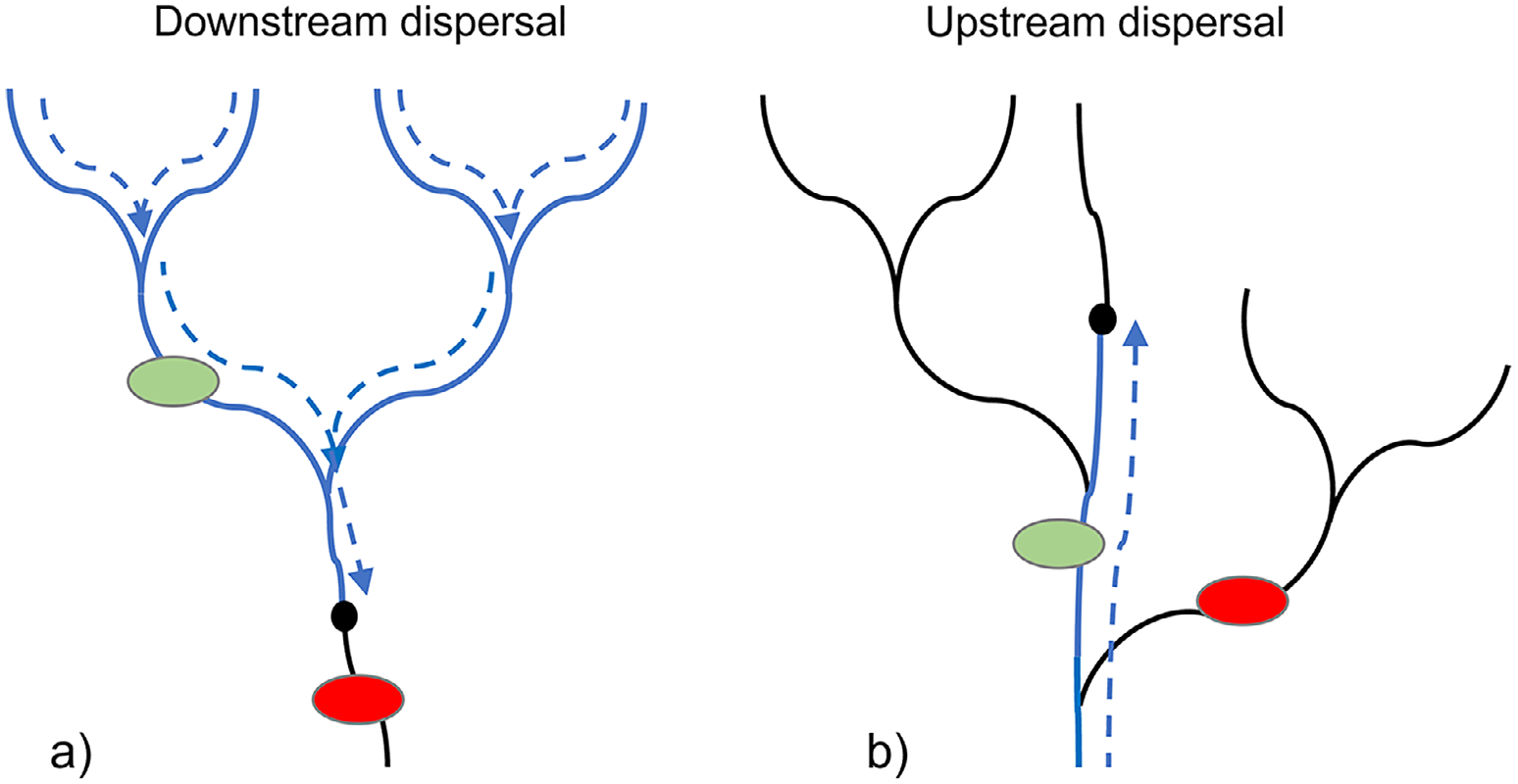
Illustration of the two dispersal pathways in a stream network used to develop the predictor variables related to dispersal limiting factors. The black circle represents a Benthic Index of Biotic Integrity (B-IBI) sample site in a stream network, represented by solid lines. The dashed lines represent the dispersal pathways used to calculate (a) predictor variables that represent the downstream dispersal to a B-IBI site from the upstream mainstem and tributaries; and (b) predictor variables that represent the upstream dispersal to a B-IBI site from the downstream mainstem only, which does not include from side tributaries and up the mainstem to a site. Note that the longitudinal origin of the analysis for downstream dispersal was from headwater streams, and for upstream dispersal, it was from an intercepting large waterbody such as the ocean or a large lake. The green and red circles illustrate how a barrier is factored into a barrier predictor variable. In this example, the green circle represents a small lake that will factor into the small lake predictor variable, while the red circle represents a small lake that will not be included in the small lake predictor.

We evaluated whether dispersal was a contributing factor explaining stream macroinvertebrate condition (B-IBI) data for some King County stream sites, including the underperforming sites. We developed predictor variables that represented three factors potentially limiting macroinvertebrate dispersal, which may be relevant to many streams in King County that are in highly urbanized settings: (1) in-stream connectivity, (2) quality of nearby source populations, and (3) quantity of habitat for up- and downstream dispersal. Quantitative measurements of these factors at the site level were unavailable. Therefore, we used geographic information system (GIS) landscape data layers from a variety of sources to develop predictor variables to represent the three factors of dispersal for inclusion in the multiple regression. The predictor variables represented two prominent pathways of aquatic invertebrate dispersal (Parkyn & Smith, 2011): (1) downstream drift, swimming, crawling, or climbing by aquatic insect larvae and aquatic adults, and (2) upstream flight by adult insects. For downstream dispersal, we calculated several metrics that included the mainstem and all tributaries upstream of each B-IBI sample site. For upstream dispersal, we calculated several metrics that only included the mainstem since it represents strictly upstream flight with respect to a B-IBI site (as opposed to flight moving down a tributary and up the mainstem to a site; Figure 3). The following sections describe these metrics.

#### In-stream connectivity

Impediments to the movement of common benthic macroinvertebrate taxa, due to anthropogenic influences or otherwise, could alter their communities (Crook et al., 2015). Dams, road-stream crossings, and lakes and wetlands (e.g., swamps/marshes) can function as barriers to the dispersal of aquatic macroinvertebrates (e.g., Bodamer & Bossenbroek, 2008; Dias et al., 2013; Richardson & Mackay, 1991; Sondermann et al., 2015). Barriers can also alter upstream connectivity and constrain species migration (e.g., Blakely et al., 2006).

We mapped the occurrence of different types of potential in-stream barriers, both natural (lakes and swamps/marshes) and artificial (dams and road-stream crossings), that could limit macroinvertebrate dispersal to a sample site. We then intersected these barriers with the National Hydrography Dataset Plus High Resolution (NHDPlusHR; USGS, 2017). The NHDPlusHR is a digital stream network at 1:24 K or better scale that provides geospatial information on streams and their related catchments across the CONUS (https://viewer.nationalmap.gov/basic). This was done using the following geospatial data layers compiled at a 1:24 K scale for every stream segment within the study area (Table 2): NHDWaterbody features from the NHDPlusHR to characterize lakes and swamps/marshes; the 2012 National Anthropogenic Barrier Dataset (Ostroff et al., 2013) to represent dams that are large enough to impede movement of biota; and the TIGER—US Census Bureau TIGER/Line Program (U.S. Census Bureau, 2019) to derive road-stream crossings by intersecting road data from the TIGER database with the NHDPlusHR stream segments in the study area. Although road-stream crossings are part of StreamCat, this feature is not calculated using the high-resolution NHDPlusHR. The scale of the study required finer resolution information on the locations of road-stream intersections, and we wanted to limit the chance of road-crossing locations being omitted from our in-stream connectivity metric if we had used medium-resolution data. The medium resolution that is available by default in StreamCat could still be useful for problems that do not require such high resolution (e.g., to identify watersheds with a high number of crossings that can be assessed further to prioritize problem crossings). Note that not all road crossings are equal impediments to movement; however, in this study, we assume that all road crossings obstruct movement equally.

We evaluated boxplot separation distances based on interquartile ranges of good and poor condition B-IBI values to determine how well each barrier type discriminated between good and poor B-IBI conditions. For each barrier type, we plotted the B-IBI condition (good or poor) against number of occurrences of a given barrier within 5 km upstream or downstream of each site. Empirical evidence suggests that most stream insects disperse over distances no greater than 5 km (Sundermann et al., 2011). We also evaluated boxplots for barriers within 1 km of each site, lest the 5-km range was too large to detect a response signal (see Appendix S1 for methods, and Appendix S2 for box plots for each barrier type). Based on our findings from these plots, we developed weighted inverse distances for use in the regression analysis. To do this, we first calculated the distance (stream, in kilometers) of a given barrier to a sample site that occurred within 5 km upstream of a site. We then took the inverse of that distance, so that the further a barrier was from a site, the less weight it had on site condition. We used the sum of inversed distances as the predictor if there was more than one barrier of a given type within 5 km upstream of a site. We repeated this for barriers within 5 km downstream of a site to include barrier predictors for both up- and downstream of sites in the regression model.

#### Quality of nearby source populations

We used a dataset of the predicted probability of each segment being in good biological condition (see Appendix S3) as a proxy for the presence of source populations upstream and/or downstream of a stream segment. We combined predicted probabilities of biological condition values for every stream segment within 5 km downstream of each site, and separately, within 5 km upstream of each site, to produce two predictors for the one model: the mean probability of good biological condition in the upstream and downstream directions, respectively.

#### Quantity of habitat for dispersal

We created predictors that represented the quantity of available stream habitat that could have a significant effect on dispersal and the macroinvertebrate communities at a site. To do this, we calculated the total stream length (in kilometers) within 5 km downstream of each sample site, and separately, within 5 km upstream of each site, and included those two predictors in the linear regression. Our calculations for the quantity of habitat for downstream dispersal included the mainstem and side tributaries, while quantity of habitat for upstream dispersal only included the mainstem (Figure 3). Due to the meandering nature of the mainstem as well as the multiple branches that could exist upstream of a site, this led to stream distances often greater than the 5 km for both upstream and downstream calculations.

### Landscape variables

From the StreamCat dataset, we included several additional landscape covariates in the multiple regression model of B-IBI condition. We used random forest analysis (Breiman, 2001) and the variable importance measure included in the R randomForest package (Liaw & Wiener, 2002) as a way of filtering hundreds of landscape predictors from StreamCat to just a small subset of predictors that explained the most variation in the response data (i.e., mean B-IBI scores). The selected StreamCat predictors represent both anthropogenic features such as impervious cover, urbanization, and agriculture, and natural features such as soil, geology, and natural land cover (e.g., forest, shrub and grassland; see Hill et al., 2016 for a complete description of catchment and watershed metrics). We used predictor variables that represented both natural and anthropogenic factors because the biological response of streams to anthropogenic activities can be influenced by a range of natural factors (Carlisle et al., 2009), such as stream size and channel gradient (Allan, 2004; Yates & Bailey, 2006). We combined several predictor variables from the StreamCat metrics to produce new variables for inclusion in the random forest analysis. For example, we used the percentage of the catchment composed of all forest types by summing existing StreamCat metrics of the National Land Cover Database (NLCD) classified as deciduous, conifer, and mixed forest land cover. In such cases, we evaluated both the new composite metrics as well as its components. We included almost 300 watershed and catchment metrics from the StreamCat dataset as predictors in the random forest models. We built a 3000-tree random forest model and identified the top predictors of mean B-IBI scores as those that had the greatest model error increase after permuting the predictor’s values (Breiman, 2001). From a list of the top 15 top predictors, a final set of predictors were selected for the multiple regression, based on having low correlation among the top variables (|*r*| < 0.70), implying that each predictor contributed unique information to the model. For two variables that were correlated, we selected the variable that had the highest variable importance measure. We considered the same metric at both watershed and catchment scales (e.g., PctForestCat and PctForestWs). However, in the final set of predictors, only one scale or the other was significant.

## RESULTS

### Final multiple regression model

We determined that four barrier types showed some ability to discriminate between good and poor B-IBI conditions within 5 and 1 km of each site based on our box plot analysis, and therefore the following barrier types were included as predictors in the multiple linear regression model: (1) large lakes (surface area ≥19 km^2^, e.g., Lake Washington), (2) small lakes (<2 km^2^), (3) road-stream crossings, and (4) swamp/marsh. From a list of top-ranked variables from the random forest analysis (see Appendix S4), we also included the following landscape predictors in the regression model (Table 2): PctForestCat2016, PctBl2016Ws, PctFrstLoss2002Cat, and SN_2008Cat.

The final multiple regression model explained more than half of the variation in mean B-IBI scores (adjusted *R*^2^ = 0.62; Table 3). The percentage of catchment composed of all forest types (PctForestCat) explained most of the variation in the model (adjusted *R*^2^ = 0.45) and was positively associated with mean B-IBI. Watershed-scale variables were tested as potential predictors in the analysis but variables at this spatial scale (including the percentage of watershed composed of all forest types; PctForestWs) did not have an important contribution to the variation explained by the model. Although our model results showed that variables related to dispersal (total stream length downstream of site, large lake downstream of site, and swamp/marsh upstream of site) were significantly associated with mean B-IBI scores, their overall contributions to the variation explained by the model were very weak and insubstantial (adjusted *R*^2^ range = 0.02–0.04; Table 3).

### Comparisons between B-IBI to IWI/ICI and B-IBI to IWI/PctForestCat

**FIGURE 4:**
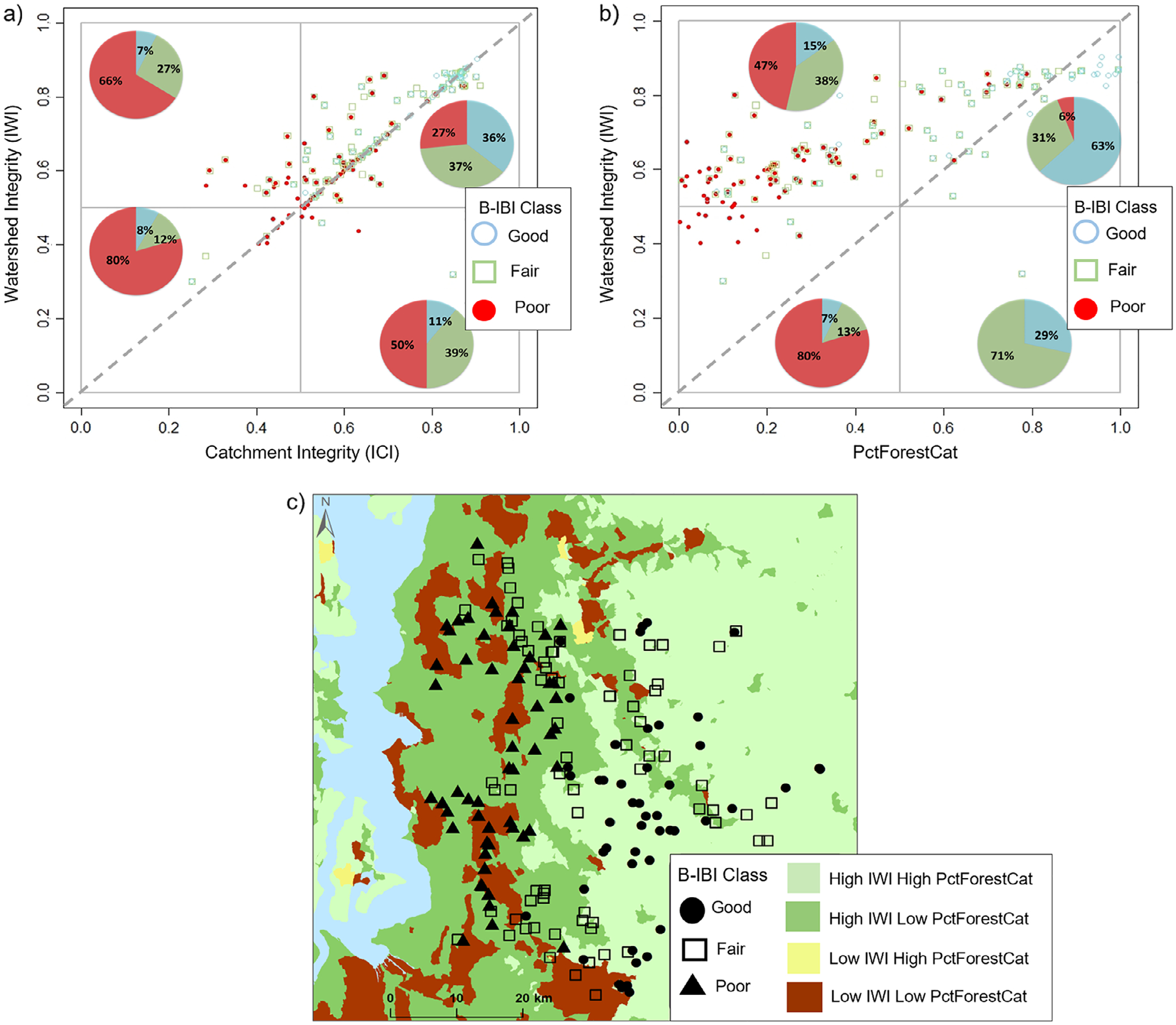
Scatterplots for King County streams and rivers relating sample site macroinvertebrate Benthic Index of Biotic Integrity (B-IBI) condition samples (*n* = 938 samples from 177 unique sites) to (a) corresponding Index of Watershed Integrity (IWI) and Index of Catchment Integrity (ICI) values and (b) corresponding IWI and PctForestCat values for each sample site, from (0) low integrity or condition to (1) high integrity or condition. Note that PctForestCat values were transferred to a scale from 0 to 1. The different colors of the plotted points represent the class of site condition from poor to good condition. The pie charts display the distribution of each B-IBI class within each quadrant. Dashed line represents the 1:1 relationship between IWI and ICI, and IWI and PctForestCat. (c) A landscape integrity map displaying the catchments of the study area according to the levels of watershed integrity and percentage forest cover in the catchments (high or low IWI and PctForestCat), with locations and mean B-IBI conditions of sample sites, from poor to good condition.

The PctForestCat, a catchment-scale variable, was the top predictor in the regression model. The scatterplots in Figure 4 show comparisons between B-IBI condition classes in relation to watershed- and catchment-scale integrity (IWI and ICI; Figure 4a), and B-IBI condition classes in relation to the IWI and PctForestCat (Figure 4b). The scatterplots indicated that most sites had high watershed and high catchment integrity (high IWI and ICI; 72% of samples, upper right quadrant, Figure 4a), of which almost equal proportions were considered by the B-IBI in good, fair, or poor condition (36%, 37%, and 27%, respectively). Replacing the catchment-scale ICI axis with PctForestCat, however, better distinguished B-IBI condition than the nationally derived ICI. Eighty-one percent of all the sites that were previously classified as underperforming (i.e., poor-condition sites in the upper right quadrant; Figure 4a) were subsequently associated with high IWI but low PctForestCat in the upper left quadrant (Figure 4b) and so were no longer classified as underperforming. In other words, many poor-condition sites with high ICI scores actually had low PctForest in the catchment (Appendix S5). The IWI/PctForestCat plot showed that most sites were from watersheds with higher integrity than found in the catchment (88% of samples above the 1:1 line) versus watersheds with lower integrity than the catchment (11.5% of samples below the line, Figure 4b). This pattern may imply that poor catchment integrity may have a more negative effect on stream condition than poor watershed integrity.

The scatterplot (Figure 4b) and integrity map (Figure 4c) revealed that of the sites from high-integrity watersheds and high-forested catchments (high IWI and PctForestCat), most were in good condition (63% of samples, upper right quadrant, Figure 4b; light green areas Figure 4c), while sites associated with high-integrity watershed but low-forested catchments (high IWI and low PctForestCat) were mostly in poor or fair condition (47% and 38% respectively, upper left quadrant, Figure 4b; dark green areas in Figure 4c). There was a small proportion of good-condition sites, however, associated with low-forested catchments (15%, upper left quadrant, Figure 4b; dark green areas in Figure 4c).

## DISCUSSION

### Operational flexibility

**FIGURE 5:**
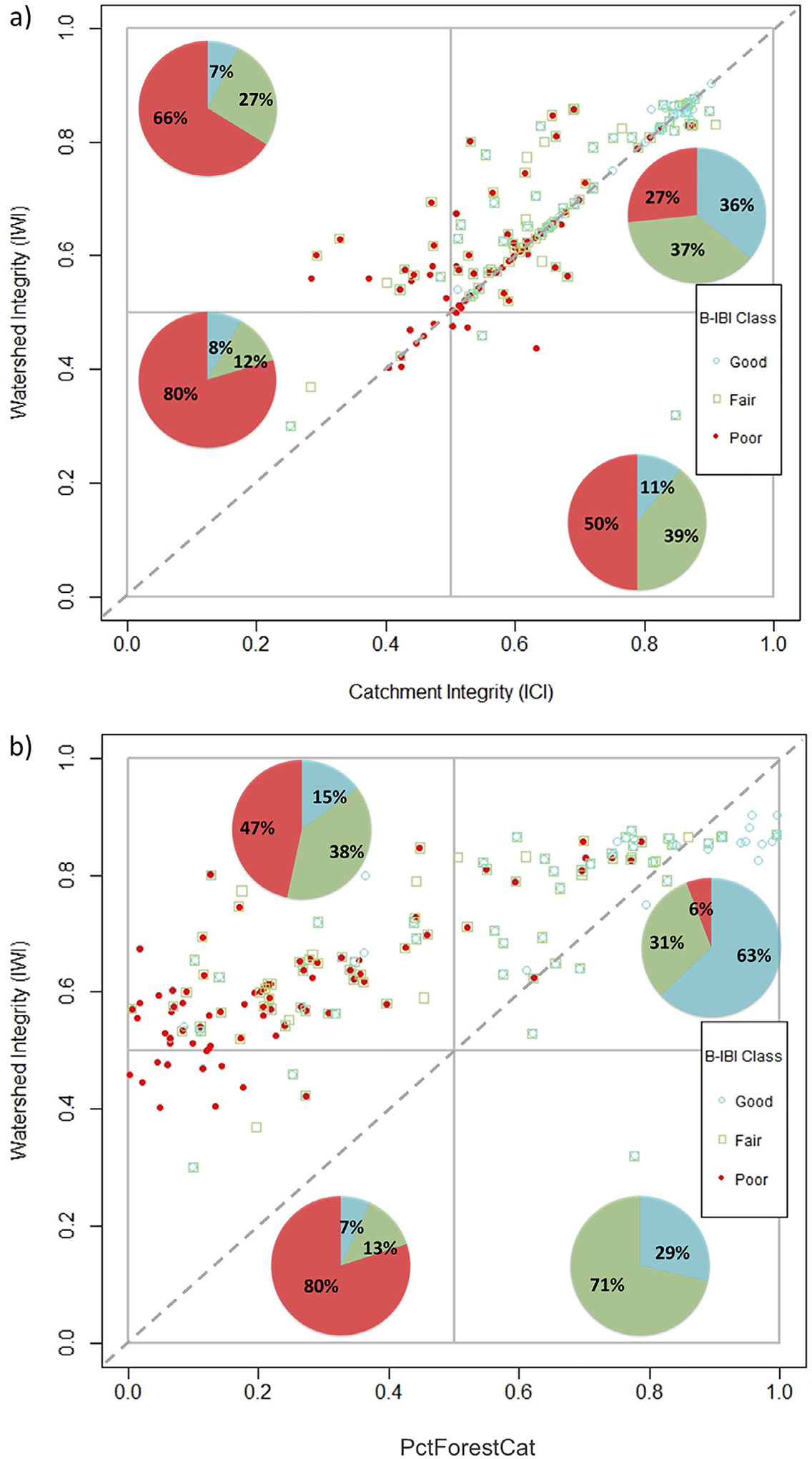
Two-dimensional scatterplots for streams and rivers across the Puget Lowland relating samples of Benthic Index of Biotic Integrity (B-IBI) condition (*n* = 203 samples from 149 sites collected by the Environmental Assessment Program at the Washington State Department of Ecology) to (a) corresponding Index of Watershed Integrity (IWI) and Index of Catchment Integrity (ICI) values and (b) corresponding IWI and PctForestCat values for each sample site, from (0) low integrity to (1) high integrity. The different colors of points denote the class of site condition from poor to good condition. The pie charts display the distribution of each B-IBI class in each quadrant. Dashed line represents the 1:1 relationship between IWI and ICI (a), and IWI and PctForestCat (b).

The multiscale approach enables analytical flexibility since these tools are not restricted to any specific dataset but are applicable to biological and landscape condition datasets of any temporal or spatial scale, in any geographical area. However, the availability of CONUS-wide data at two scales provides default data that can be used in instances where such data are not locally available, although as we have shown here, the data may (as in the case of the IWI) or may not (as in the case of the ICI) be well-suited to particular applications. In this way, the multiscale approach could benefit conservation planning in any part of the world undergoing intense urban growth, provided there is access to biological data and landscape condition (or stressor) data at multiple spatial scales (e.g., catchment and watershed scales). Riato et al. (2020) demonstrated the flexibility of the multiscale approach by using stream biological condition data from different regions and states across the United States, including the Central Appalachian region, the high gradient region of New Jersey and Connecticut, for fish, macroinvertebrates, and diatoms assessed by a variety of tools (e.g., the Biological Condition Gradient, and the B-IBI). For the landscape integrity data, they applied the nationally available landscape integrity indicators, the IWI and ICI, and state- and regional-derived watershed and stream-reach-scale integrity indicators. In this paper, we also illustrate the flexibility of the approach by incorporating a different dataset from stream sites sampled across the Puget Lowland by another agency, the Washington Department of Ecology (Figure 5).

The multiscale approach is not limited to urban settings like our case study of King County; it could be applied to any type of setting to support conservation-related decisions. For example, Riato et al. (2020) demonstrated the utility of the multiscale approach in the Central Appalachian, where two-thirds of the land use is forested and the remaining portion is mostly agriculture with some mining. In this case study example, we demonstrate the importance of incorporating regionally relevant stressors in the scatterplot or map. In our Central Appalachian case study (Riato et al., 2020), around 50% of poor-condition sites were associated with high watershed and high catchment integrity (high IWI/ICI) in a scatterplot. When we included a regionally important indicator that represented measures of conductivity from the water, which is a major stressor from mining impacts in that region, half of those poor-condition sites that had high watershed and high catchment integrity were related to elevated conductivity in the water mostly from mining.

In the same region, Riato et al. (2020) also illustrated how the multiscale approach could help managers identify the spatial scale(s) that stream biological condition is most responsive to. Riato et al. (2020) related three spatial scales of landscape integrity information (watershed, catchment, and reach scale) to stream macroinvertebrate condition data in a 3D scatterplot. In this case, the biological degradation in these streams was likely due to conditions at the reach scale. Identifying the spatial scale(s) that stream biological condition is most responsive to could enable managers to focus on the scale(s) in which restoration activities are more likely to succeed.

### Regionally important indicator

We expected to find that dispersal, one of our main hypotheses, was a contributing factor explaining stream macroinvertebrate condition (B-IBI) data for some King County stream sites, including the underperforming sites. The results of the multiple linear regression model, however, showed that dispersal limiting factors did not relate well to B-IBI scores. Our analysis therefore does not support the hypothesis that limited dispersal is a factor affecting most catchments in the study, although the fact that the indicators show up as significant variables in the regression shows that dispersion may play some limited role. The relatively weak effect of our dispersal indices on B-IBI could be due to our use of the following: (1) B-IBI scores which, although highly influenced by taxa richness, do not reflect specific taxa, and, as such, may be too coarse of a biological measure to relate to dispersal; using presence/absence of specific taxa may be more biologically appropriate; and (2) GIS data as proxies for direct measurements of dispersal. Other studies, however, have shown GIS-based indicators of dispersal to be effective. GIS-based indicators, for example, have been used to develop a connectivity index for river and stream macroinvertebrates in South Africa based on in-stream barriers and land cover fragmentation (Rivers-Moore et al., 2016); to model the effect of in-stream and terrestrial barriers on the dispersal of aquatic insect species in a Central European mountain catchment (Sondermann et al., 2015); and as a proxy for indicating habitat suitability and barriers for dispersing aquatic insects (Heino et al., 2017). Hence the use of GIS data to represent dispersal may not be an issue here. While the results of our GIS analysis indicate dispersal is not an important factor influencing B-IBI scores, it would be desirable to verify these results using direct measurements of dispersal. We note, however, that such data are hard and costly to obtain and verification may not be feasible for many management programs.

Application of prioritization tools at regional scales requires adaptation to consider regionally important stressors. For the King County area, we found that a catchment-scale indicator related to forest condition, PctForestCat, explained most of the variation in mean B-IBI scores in the final multiple linear regression model (adjusted *R*^2^ = 0.45). Our findings corroborate other studies in the Puget Lowland that showed degraded stream condition, and negative impacts to B-IBI scores were best explained by large-scale stressors associated with forest loss and urbanization over larger spatial scales rather than local stressors (DeGasperi et al., 2009; King County, 2019). The transformation of forest to developed areas has led to excess stormwater flows across the watershed, causing bank erosion and slope failure, and consequently, excess sediment deposits within the stream that can seriously impact macroinvertebrate communities (Aho, 2011; Kitsap County, 2008). It is therefore likely that there are multiple, related factors correlated with the PctForestCat metric that may be explaining the variation in mean B-IBI scores. Our methods used to develop an effective catchment-scale indicator for King County based on nationwide datasets in StreamCat could provide an important tool for conservation and management of stream ecosystems across the CONUS, especially in data-limited areas. To summarize our methods: (1) plot the national IWI and ICI values associated with stream sites, where the colored points represent site biological condition; (2) conduct a regression analysis to identify an important regional variable like PctForestCat; (3) replace the IWI and/or ICI (in this case the ICI) in the scatterplot with the regional variable if appropriate.

This study highlights how unexpected behaviors in site condition can lead to insights into the causes of degraded conditions. Riato et al. (2020) described how managers could use the IWI and ICI in a scatterplot and landscape integrity map to identify high-priority sites for restoration and protection (Table 1). Based on this previous work, we expected good-condition sites to generally fall within the upper right quadrant and poor-condition sites within the lower two quadrants. However, for streams in King County, we found that most sites in poor condition fell in the upper right quadrant (Figure 4a). This misalignment occurred possibly because the original IWI/ICI framework was unsuitable for this area, and that further investigation was required to identify local-scale factor(s) associated with poor condition that was not accounted for by the landscape integrity indicators. In doing so, this could provide more informed management decisions on where and how to protect or restore stream habitat. Indeed, we found that by substituting the ICI with a more regionally derived and focused catchment-scale indicator of forest condition (PctForestCat), which contained information not represented in the ICI, it explained most of the misalignment between poor condition and integrity at the watershed and catchment scale (Figure 4b). Most of the poor-condition sites that appeared to be underperforming because they were in the upper right quadrant (i.e., high-integrity watershed/high-integrity catchment) based on the IWI/ICI scatterplot, subsequently, were classified as poor-condition sites in the upper left quadrant (i.e., high-integrity watershed/poor-condition catchment) of the IWI/PctForestCat scatterplot, and so were no longer classified as underperforming. For the underperforming sites that could not be explained by the ICI or PctForestCat (upper right quadrant, Figure 4a,b), biological degradation in these streams may be due to a variety of stressors at a more localized scale not captured in the geospatial layers that make up the ICI or PctForestCat (e.g., local pollutants from urban stormwater). Although PctForestCat is not measured directly within the ICI, information on forest condition may be captured in the ICI by other stressor metrics (e.g., the forest cover loss metric; tab. 1 in Thornbrugh et al., 2018). However, the ICI likely did not reflect forest condition for this study area probably because the ICI is an aggregation of six different indices that considers a whole host of other stressors within the catchment. The effects of forest condition would therefore be diluted by the additional metrics that can render the ICI insufficient for this study area.

We observed similar trends using a different dataset of B-IBI scores from stream sites sampled across the Puget Lowland by another agency, the Washington Department of Ecology (Appendix S6), where many supposed underperforming sites in the upper right quadrant of the IWI/ICI scatterplot were poor-condition sites in poor-condition catchments in the upper left quadrant of the IWI/PctForestCat plot (Figure 5). These results suggest that PctForestCat is important not just within the King County area, but the entire Puget Lowlands ecoregion.

Good-condition sites were related to high PctForestCat values overall, and fair- and poor-condition sites to low PctForestCat values (Figure 4a,b), implying that the PctForestCat metric can discriminate good sites from bad, which is the ultimate test of the effectiveness of a metric in biological assessments (Stoddard et al., 2008). Nevertheless, there were several good-condition sites associated with low-forested catchments (upper left quadrant, Figure 4b; dark green areas, Figure 4c). One possible explanation for this result is that other environmental factors in the catchment or at a more local scale were influencing the response of B-IBI condition at these good-condition sites that were not represented by the PctForestCat metric. For example, some of those sites, although located in an agricultural setting, had a relatively healthy riparian zone. Riparian vegetation is known to enhance stream macroinvertebrate communities by providing organic matter and shading (e.g., Hawkins et al., 1982; Hession et al., 2000). The strong association between riparian vegetation and aquatic macroinvertebrate assemblages could be a factor that is influencing the response of B-IBI condition at these good-condition sites although this hypothesis requires further testing. Another possible explanation is that there is relatively good condition upstream beyond the 5 km examined here, which allows for some dispersal downstream that could partially mask the impacts of local catchment conditions.

It is also worth noting that there were two good-condition sites within the lower left quadrant associated with low-integrity/-condition watersheds and catchments (i.e., overperforming sites; Figure 4a,b). Since only two sites misaligned, this suggests that the sites were correctly classified as overperforming, but in a scenario where there are many overperforming sites, this might indicate that one or more of the landscape indicators were insufficient.

Although the ICI was not a suitable catchment-scale indicator for King County, Riato et al. (2020) demonstrated the utility of the national ICI in stream condition assessments in different parts of the United States (e.g., the high gradient region of New Jersey and the state of Connecticut). In that respect, the national IWI and ICI, and landscape indicators derived from the national StreamCat dataset such as the PctForestCat, could provide important indicators to support conservation management and decision-making across the United States at local or larger regional scales.

### Prioritizing and optimizing conservation actions

While conservation prioritization is widely researched and commonly applied, approaches for conservation in urban landscapes are relatively understudied (Ettinger et al., 2021). Yet prioritizing conservation action in highly urbanized areas and those undergoing urbanization has become increasingly important with the rapid growth of urban areas worldwide, which has led to a decline in habitat quantity, quality, and connectivity, and a subsequent loss of biodiversity (Seto et al., 2012; Syphard et al., 2011). Moreover, deciding where to best direct resources to conservation efforts is more challenging in cities and their surrounds, where urban green spaces often compete with other uses, such as housing (Colding et al., 2020). In light of this, further development of decision support frameworks and tools for conservation prioritization in urbanized settings is imperative. To address this need, recent initiatives have developed landscape models to prioritize stream protection, restoration, and management actions across heavily urbanized watersheds in the state of California based on stream bioassessment data and multiscale landscape stressors from the StreamCat dataset (e.g., Beck et al., 2019, 2020; Stein et al., 2022). Beck et al. (2019, 2020) examined “underperformance” as a way to prioritize sites for restoration. A simple multiscale landscape framework, such as the one presented here, could provide important spatial information by way of scatterplots and landscape integrity maps for prioritizing conservation action in rapidly urbanizing areas, as illustrated in our case study of King County. PctForestCat could be helpful for setting conservation priorities, although this tool could de facto give sites in urban areas a low priority because of the negative correlation that generally exists between forests and urban areas. While we acknowledge the importance of prioritizing restoration or protection efforts so that managers get the biggest “bang for one’s buck” biologically, this is only one piece of information when considering which sites to conserve. Other pieces of information for consideration could include social, economic, and environmental justice factors. For example, sites that may be lower prioritized for protection with this tool may be prioritized highly because they provide green space that reduces heat-related stress on underserved communities (Kabisch & van den Bosch, 2017). Note that the multiscale approach does not prioritize sites within a quadrant or within a catchment type in the map by individually ranking each site in order of priority.

Our approach is a straightforward way of integrating landscape information at multiple spatial scales that is not meant to replace but complement site-specific information; it could also complement integrated landscape analysis by allowing other scales of data to be included. These visual tools could support economically optimal management decisions and effectively convey those decisions to stakeholders; the graphical nature of the scatterplot and landscape integrity map enables identification and prioritization of sites for restoration and protection, as well as identification of areas where remediation efforts would be more costly and less effective. For example, in the scatterplot and map that relates the B-IBI condition of King County stream sites to the IWI and PctForestCat, we could identify sites associated with high IWI and high PctForestCat values (IWI and PctForestCat values >0.5 in upper right quadrant of Figure 4b; and light green catchments in Figure 4c), as having the most restoration or protection potential. In this highly urbanized area, restoration efforts to improve stormwater control to reduce fine sediment inputs to the stream channel, or conservation efforts to protect forests and limit development to maintain stream functions (King County, 2019), would be less costly (although actual project costs or savings would have to be determined by comparative economic analyses) and have a higher probability of success if those efforts target existing, high-integrity infrastructure that is more capable of sustaining them (e.g., areas that have forested land and less development). Managers could reduce the risk of directing limited funds to suboptimal sites in large deforested areas that require great effort to mitigate the effects of forest removal and urban development (i.e., IWI values <0.5 in the lower two quadrants, Figure 4b; yellow and brown catchments in Figure 4c). In this way, such a scatterplot and map allow for prioritizing and optimizing specific management actions, which are critical steps in conservation planning (Erős et al., 2018; Tallis et al., 2021). In addition, these tools could be extremely effective in conservation planning and management because of their ease of use by resource managers and transparency to stakeholders.

## CONCLUDING REMARKS

Simple approaches that integrate biological condition data with multiscale landscape integrity information, such as presented here, provide an easily implemented way of prioritizing candidate streams for restoration or protection at local or large geographic scales, and support the growing need for integrating landscape information into freshwater conservation, management, and decision-making. More importantly, this simple approach is most effective when regionally appropriate stressors are incorporated into the multiscale landscape framework.

## Supplementary Material

Supplement1

Supplement2

Supplement3

Supplement4

Supplement5

Supplement6

## Figures and Tables

**TABLE 1:** Summary of the various possible management applications of the quadrants in a scatterplot and catchment classes in a landscape integrity map to support possible stream restoration and protection actions.

Existing landscape integrity and site condition				Outcome	
Scatterplot quadrant site is within	Landscape integrity map catchment class site is within	Level of watershed/catchment integrity	Biological condition of site	Possible explanation of site condition	Possible management action
Upper right	Light green	Watershed: High Catchment: High	Poor (i.e., underperforming relative to their catchment and watershed integrity)	Poor performance is likely due to low integrity at the site-scale.	High-priority site for restoration because site-level restoration would likely be feasible and effective if watershed and catchment integrity are good.
Upper right	Light green	Watershed: High Catchment: High	Good	Good performance is likely due to high integrity at the reach, catchment and watershed scale.	High-priority site for protection because site-level protection would likely be feasible and effective if watershed and catchment integrity are good.
Upper left	Dark green	Watershed: High Catchment: Low	Poor	Poor performance is likely due to low integrity at the catchment scale.	Possible opportunity for catchment-level restoration (if financial resources can support these efforts at that scale) because these efforts would likely be feasible and effective if watershed integrity is good.
Upper left	Dark green	Watershed: High Catchment: Low	Good	Good performance could be due to other environmental factors in the catchment or at a more local scale not represented by the landscape indicator.	Possible opportunity for site-level protection because these efforts would likely be feasible and effective if watershed and catchment integrity are good. Gaining information on those factors first would be necessary to determine its feasibility and effectiveness.
Lower left or right	Brown or yellow	Catchment: Low and/or Watershed: Low	Poor	Poor performance is likely due to catchment and/or watershed degradation, as well as site-specific factors.	Restoration of poor-condition sites unlikely to succeed in poor landscapes; choose poor sites in the upper quadrants in which to invest in more feasible and effective restoration efforts.
Lower left or right	Brown or yellow	Catchment: Low and/or Watershed: Low	Good (i.e., overperforming relative to their catchment and/or watershed integrity)	Good performance could be due to other environmental factors in the catchment or at a more local scale not represented by the landscape indicator.	Protection of good-condition sites unlikely to succeed in poor landscapes; direct resources to good-condition sites in the upper quadrants where it should be less costly and likely effective to protect.

*Note*: These descriptions assume that the watershed and catchment indicators perform properly as indicators of integrity at their respective scales. Management actions are suggested based on how site restoration or protection would respond given the watershed and catchment integrity, without taking into account any other factors such as economic and social costs.

**TABLE 2:** List and description of predictor variables for multiple linear regression representing different factors known to influence stream biological condition.

Predictor variable category	Variable name	Description	Data source	Reference
In-stream connectivity	Large lakes downstream	Natural barrier; lake surface area ≥19 km^2^; large lakes located downstream of sites only (not upstream)	2017 NHDPlusHR	USGS (2017)
	Small lakes downstream	Natural barrier; lake <2 km^2^; there were no lakes between 2 and 19 km^2^	2017 NHDPlusHR	USGS (2017)
	Small Lakes upstream	Natural barrier; lake <2 km^2^; there were no lakes between 2 and 19 km^2^	2017 NHDPlusHR	USGS (2017)
	Swamp/marsh downstream	Natural barrier; mostly palustrine shrub–scrub type <1 km^2^	2017 NHDPlusHR	USGS (2017)
	Swamp/marsh upstream	Natural barrier; mostly palustrine shrub–scrub type <1 km^2^	2017 NHDPlusHR	USGS (2017)
	Road-stream crossing downstream	Artificial barrier	2019 TIGER—US Census Bureau TIGER/Line Program	US Census Bureau (2019)
	Road-stream crossing upstream	Artificial barrier	2020 TIGER—US Census Bureau TIGER/Line Program	US Census Bureau (2019)
Quality of nearby source populations	Mean prG_BMMI of upstream reaches	Predicted probability that a stream segment is in good biological condition based on a random forest model of the USEPA’s National Rivers and Streams Assessment benthic invertebrate multimetric index (BMMI)	StreamCat	Hill et al. (2017)
	Mean prG_BMMI of downstream reaches	Predicted probability that a stream segment is in good biological condition based on a random forest model of the USEPA’s National Rivers and Streams Assessment BMMI	StreamCat	Hill et al. (2017)
Quantity of habitat for upstream or downstream dispersal	Total stream length of downstream reaches	Total length of stream segment (in kilometers) within 5 km of site	2017 NHDPlusHR	USGS (2017)
	Total stream length of upstream reaches	Total length of stream segment (in kilometers) within 5 km of site	2017 NHDPlusHR	USGS (2017)
Landscape	PctForestCat2016	Percentage of catchment composed of all forest types derived by summing existing StreamCat metrics of National Land Cover Database (NLCD) 2016 data classified as deciduous, conifer and mixed forest land cover	StreamCat	Hill et al. (2016)
	PctBl2016Ws	Percentage of watershed area classified as barren land cover (NLCD 2016)	StreamCat	Hill et al. (2016)
	PctFrstLoss2002Cat	Percent Forest cover loss (tree canopy cover change) for 2002 within catchment	StreamCat	Hill et al. (2016)
	SN_2008Cat	Annual gradient map of precipitation-weighted mean deposition for average sulfur and nitrogen wet deposition for 2008 in kilograms of S + N/ha/yr, within catchment	StreamCat	Hill et al. (2016)

*Note*: Except for landscape variables, all variables are calculated within 5 km upstream or downstream of a B-IBI site. Note that calculations of variables upstream of site include data associated with the mainstem (relative to the site) and tributary inflows to represent the downstream dispersal pathway via drift, swimming, crawling, or climbing by aquatic insect larvae and aquatic adults. Calculations of variables downstream of site include data associated with the mainstem only since it represents strictly upstream flight by adult insects (see Figure 3 for more details).

Abbreviations: B-IBI, Benthic Index of Biotic Integrity; NHDPlusHR, National Hydrography Dataset Plus High Resolution; USEPA, U.S. Environmental Protection Agency.

**TABLE 3:** Multiple linear regression model results for King County dataset. The response variable is the mean Benthic Index of Biological Integrity (B-IBI) score (*n* = 177) based on samples collected from 2012 to 2019; all predictor variables and model are significant at an α of 0.05.

Final model predictor variables	Direction of response to predictor variables	Variation in response explained by individual predictors (adjusted *R*^2^)	Model fit (adjusted *R*^2^)
PctForestCat	Positive	0.45	0.62
PctBlWs	Negative	0.02	
Swamp/marsh upstream^a^	Positive	0.04	
Large lake downstream^a^	Negative	0.02	
Total stream length (km) of downstream reaches	Positive	0.03	

*Note*: PctForestCat is the percentage of catchment composed of all National Land Cover Database forest types; PctBlWs is the percentage of watershed area classified as barren land cover.

a Sum of inversed distances (in kilometers) of a given barrier within 5 km of a site.

## Data Availability

Code (Weber, 2022) is available from Zenodo: https://doi.org/10.5281/zenodo.7275096. Data (King County, 2020b) are available from the Puget Sound Stream Benthos site: https://pugetsoundstreambenthos.org/Biotic-Integrity-Scores.aspx.
