# Supplementary material for: A multiscale landscape approach for prioritizing river and stream protection and restoration actions
Source: Ecosphere. Author manuscript; Available in PMC 2024 Jan 19. (PMC9903358; doi:10.1002/ecs2.4350)
Supplement: Supplement1 [file NIHMS1868745-supplement-Supplement1.docx]

**A multiscale landscape approach for prioritizing river and stream protection and restoration actions**

***Ecosphere***

Luisa Riato^1^, Scott G. Leibowitz^2^, Marc H. Weber^2^, Ryan A. Hill^2^

1. Oak Ridge Institute for Science and Education (ORISE) Post-Doctoral Fellow c/o U.S. Environmental Protection Agency, Center for Public Health and Environmental Assessment, Pacific Ecological Systems Division, 200 SW 35^th^ St., Corvallis, OR 97333 USA; [riato.luisa@epa.gov](mailto:riato.luisa@epa.gov)
2. U.S. Environmental Protection Agency, Center for Public Health and Environmental Assessment, Pacific Ecological Systems Division, 200 SW 35^th^ St., Corvallis, OR 97333 USA; leibowitz.scott@epa.gov, weber.marc@epa.gov, hill.ryan@epa.gov

**Appendix S4. Top ranked variables from the random forest analysis for predicting B-IBI condition in King County, Washington State, United States. Mean Decrease Accuracy (%IncMSE) is the normalized difference of the classification accuracy for the observations excluded from model calibration, and the classification accuracy for the same observations when values of the predictor are randomly permuted. The higher the mean decrease in accuracy, the more important the predictor is to the model. A description of each landscape metric and how they were derived is provided here: https://gaftp.epa.gov/epadatacommons/ORD/NHDPlusLandscapeAttributes/StreamCat/Documentation/DataDictionary.html**

| **Predictor Variable** | **%IncMSE** |
| --- | --- |
| PctForest2016Cat | 40.7 |
| PctForest2016CatRp100 | 35.8 |
| SN_2008Cat | 34.7 |
| MSST_2014 | 32.6 |
| NO3_2008Cat | 30.3 |
| PctDecid2016WsRp100 | 28.9 |
| PctConif2016Cat | 28.5 |
| PctFrstLoss2002Cat | 27.2 |
| PctShrb2016Ws | 26.0 |
| PctBl2016Ws | 25.9 |
| NH4_2008Cat | 23.0 |
| InorgNWetDep_2008Cat | 22.7 |
| PctMxFst2013CatRp100 | 22.2 |
| PctShrb2013CatRp100 | 21.9 |
| PctMxFst2016CatRp100 | 21.4 |
