# Supplementary material for: A multiscale landscape approach for prioritizing river and stream protection and restoration actions
Source: Ecosphere. Author manuscript; Available in PMC 2024 Jan 19. (PMC9903358; doi:10.1002/ecs2.4350)

**A multiscale landscape approach for prioritizing river and stream protection and restoration actions**

***Ecosphere***

Luisa Riato^1^, Scott G. Leibowitz^2^, Marc H. Weber^2^, Ryan A. Hill^2^

1. Oak Ridge Institute for Science and Education (ORISE) Post-Doctoral Fellow c/o U.S. Environmental Protection Agency, Center for Public Health and Environmental Assessment, Pacific Ecological Systems Division, 200 SW 35^th^ St., Corvallis, OR 97333 USA; [riato.luisa@epa.gov](mailto:riato.luisa@epa.gov)
2. U.S. Environmental Protection Agency, Center for Public Health and Environmental Assessment, Pacific Ecological Systems Division, 200 SW 35^th^ St., Corvallis, OR 97333 USA; leibowitz.scott@epa.gov, weber.marc@epa.gov, hill.ryan@epa.gov

**Appendix S5. Scatterplot for King County streams and rivers relating sample site macroinvertebrate Benthic Index of Biotic Integrity (B-IBI) condition samples (n=938 samples from 177 unique sites) to corresponding PctForestCat and ICI values for each sample site, from (0) low integrity or condition to (1) high integrity or condition. The different color of the plotted points represents the class of site condition from poor (red) to good (blue) condition. Dashed line represents the 1:1 relationship between PctForestCat and ICI.**


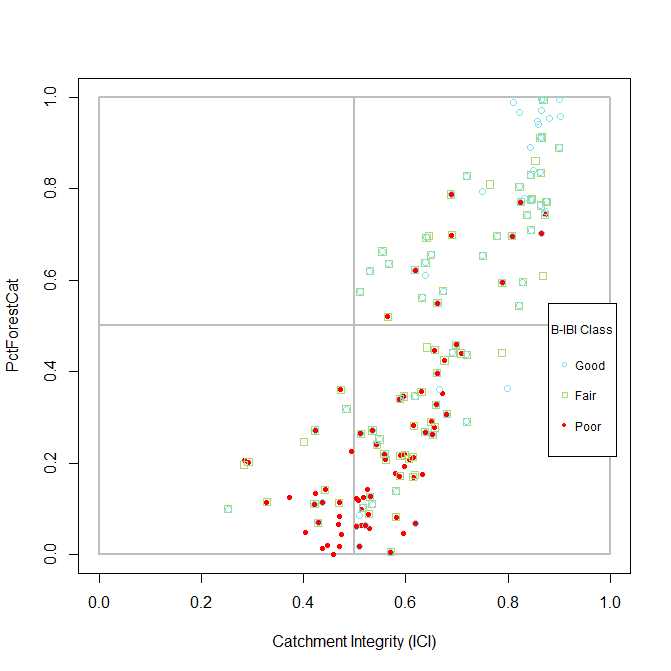

Supplement: Supplement2 [file NIHMS1868745-supplement-Supplement2.docx]
