# Supplementary material for: A multiscale landscape approach for prioritizing river and stream protection and restoration actions
Source: Ecosphere. Author manuscript; Available in PMC 2024 Jan 19. (PMC9903358; doi:10.1002/ecs2.4350)
Supplement: Supplement3 [file NIHMS1868745-supplement-Supplement3.docx]

**A multiscale landscape approach for prioritizing river and stream protection and restoration actions**

***Ecosphere***

Luisa Riato^1^, Scott G. Leibowitz^2^, Marc H. Weber^2^, Ryan A. Hill^2^

1. Oak Ridge Institute for Science and Education (ORISE) Post-Doctoral Fellow c/o U.S. Environmental Protection Agency, Center for Public Health and Environmental Assessment, Pacific Ecological Systems Division, 200 SW 35^th^ St., Corvallis, OR 97333 USA; [riato.luisa@epa.gov](mailto:riato.luisa@epa.gov)
2. U.S. Environmental Protection Agency, Center for Public Health and Environmental Assessment, Pacific Ecological Systems Division, 200 SW 35^th^ St., Corvallis, OR 97333 USA; leibowitz.scott@epa.gov, weber.marc@epa.gov, hill.ryan@epa.gov

**Appendix S6. Description of Puget Lowland macroinvertebrate benthic index of biotic integrity (B-IBI) dataset.**

The Benthic Index of Biotic Integrity (B-IBI) is routinely used to assess the biological condition of streams across the Puget Lowland. The Puget Lowland dataset (n=203 samples) comprised of B-IBI samples scores with at least 300 counts with a target count of 500, from 149 unique wadeable and non-wadeable stream sites collected by the Environmental Assessment Program at the Washington State Department of Ecology (<https://ecology.wa.gov/Research-Data/Monitoring-assessment/River-stream-monitoring/Habitat-monitoring/Stream-biological-monitoring>). Streams were sampled once per year between July and October from 2009 to 2018 following standard protocols (Larson et al. 2019). Data and details of the assessment are available at: <https://www.pugetsoundstreambenthos.org>. We categorized samples into good, fair, and poor biological condition based on condition class thresholds for B-IBI scores in the Western WA region where the sample sites were located (one of three regions in Washington defined by precipitation gradients; Larson et al. 2019). B-IBI scores ≤ 49.98 were classified as poor, scores ≥ 73.73 were classified as good, and between 49.98 and 73.73 were fair.

**References**

Larson, C. A., G. Merritt, J. Janisch, J. Lemmon, M. Rosewood-Thurman, B. Engeness, S. Polkowske, and G. Onwumere. 2019. The first statewide stream macroinvertebrate bioassessment in Washington State with a relative risk and attributable risk analysis for multiple stressors. Ecological Indicators 102:175–185.
