# Supplementary material for: A multiscale landscape approach for prioritizing river and stream protection and restoration actions
Source: Ecosphere. Author manuscript; Available in PMC 2024 Jan 19. (PMC9903358; doi:10.1002/ecs2.4350)
Supplement: Supplement4 [file NIHMS1868745-supplement-Supplement4.docx]

**A multiscale landscape approach for prioritizing river and stream protection and restoration actions**

***Ecosphere***

Luisa Riato^1^, Scott G. Leibowitz^2^, Marc H. Weber^2^, Ryan A. Hill^2^

1. Oak Ridge Institute for Science and Education (ORISE) Post-Doctoral Fellow c/o U.S. Environmental Protection Agency, Center for Public Health and Environmental Assessment, Pacific Ecological Systems Division, 200 SW 35^th^ St., Corvallis, OR 97333 USA; [riato.luisa@epa.gov](mailto:riato.luisa@epa.gov)
2. U.S. Environmental Protection Agency, Center for Public Health and Environmental Assessment, Pacific Ecological Systems Division, 200 SW 35^th^ St., Corvallis, OR 97333 USA; leibowitz.scott@epa.gov, weber.marc@epa.gov, hill.ryan@epa.gov

**Appendix S3. Description and use of dataset of predicted probabilities of stream condition across the conterminous United States.**

Dispersal and establishment of healthy biological assemblages depends not only on the degree of connectivity between a sample site and nearby stream segments, but also on the presence of source populations nearby (Sondermann et al. 2015). Hence, a stream network could be barrier free, but some segments could still limit the pool of available source populations because of poor habitat quality (Diebel et al. 2015). We used a dataset of the predicted probability of each segment being in good biological condition as a proxy for the presence of source populations upstream and/or downstream of a stream segment. Predictions of biological condition were modeled based on a benthic invertebrate multimetric index (MMI) from the USEPA’s National Rivers and Streams Assessment as a response variable and landscape information taken from the StreamCat dataset (Hill et al. 2016) as predictor variables (Hill et al. 2017). We expanded the MMI model, originally developed to estimate condition only at perennial streams, to predict biological condition for non-perennial streams, to provide values for all stream segments in our study area. Finally, we combined MMI values for every stream segment within 5 km downstream of each site, and separately, within 5 km upstream of each site, to produce two predictors for the one model; the mean probability of good MMI in the upstream and downstream directions, respectively. Note that if only part of a stream reach was contained within the 5 km radius, we still included the entire MMI value for that reach.

**References**

Diebel, M. W., M. Fedora, S. Cogswell, and J. R. O’Hanley. 2015. Effects of Road Crossings on Habitat Connectivity for Stream-Resident Fish. River Research and Applications 31:1251–1261.

Hill, R. A., E. W. Fox, S. G. Leibowitz, A. R. Olsen, D. J. Thornbrugh, and M. H. Weber. 2017. Predictive mapping of the biotic condition of conterminous U.S. rivers and streams. Ecological Applications 27:2397–2415.

Hill, R. A., M. H. Weber, S. G. Leibowitz, A. R. Olsen, and D. J. Thornbrugh. 2016. The Stream-Catchment (StreamCat) Dataset: A Database of Watershed Metrics for the Conterminous United States. JAWRA Journal of the American Water Resources Association 52:120–128.

Sondermann, M., M. Gies, D. Hering, M. Schröder, and C. K. Feld. 2015. Modelling the effect of in-stream and terrestrial barriers on the dispersal of aquatic insect species: a case study from a Central European mountain catchment. Fundamental and Applied Limnology / Archiv für Hydrobiologie 186:99–115.
