# Supplementary material for: A multiscale landscape approach for prioritizing river and stream protection and restoration actions
Source: Ecosphere. Author manuscript; Available in PMC 2024 Jan 19. (PMC9903358; doi:10.1002/ecs2.4350)
Supplement: Supplement5 [file NIHMS1868745-supplement-Supplement5.docx]

**A multiscale landscape approach for prioritizing river and stream protection and restoration actions**

***Ecosphere***

Luisa Riato^1^, Scott G. Leibowitz^2^, Marc H. Weber^2^, Ryan A. Hill^2^

1. Oak Ridge Institute for Science and Education (ORISE) Post-Doctoral Fellow c/o U.S. Environmental Protection Agency, Center for Public Health and Environmental Assessment, Pacific Ecological Systems Division, 200 SW 35^th^ St., Corvallis, OR 97333 USA; [riato.luisa@epa.gov](mailto:riato.luisa@epa.gov)
2. U.S. Environmental Protection Agency, Center for Public Health and Environmental Assessment, Pacific Ecological Systems Division, 200 SW 35^th^ St., Corvallis, OR 97333 USA; leibowitz.scott@epa.gov, weber.marc@epa.gov, hill.ryan@epa.gov

**Appendix S2. Boxplots showing the B-IBI condition category (Good or Poor) against the number of occurrences of a) small lakes within 5 km upstream, b) small lakes within 1 km upstream, c) swamps within 5 km upstream, d) swamps within 1 km upstream, e) road-stream crossings within 5 km upstream, f) road-stream crossings within 1 km upstream, g) large lakes within 5 km downstream, h)** **large lakes within 1 km downstream, i) small lakes within 5 km downstream, j) small lakes within 1 km downstream, k)** **swamps within 5 km downstream, l) swamps within 1 km downstream, m) road-stream crossings within 5 km downstream, and n) road-stream crossings within 1 km downstream. Boxplots also show** **B-IBI condition category (Good or Poor) against o) total stream length (km) of upstream reaches within 5 km, p) total stream length (km) of upstream reaches within 1 km, q) total stream length (km) of downstream reaches within 5 km, and r) total stream length (km) of downstream reaches within 1 km. Lines in boxes represent the medians, boxes represent the interquartile ranges (25th and 75th percentiles), whiskers represent 1.5 interquartile ranges, and the dots represent outliers. Note that there are no boxplots showing the B-IBI condition category against the number of occurrences for large lakes within 5 km or 1 km upstream because there were no large lakes present upstream.** **
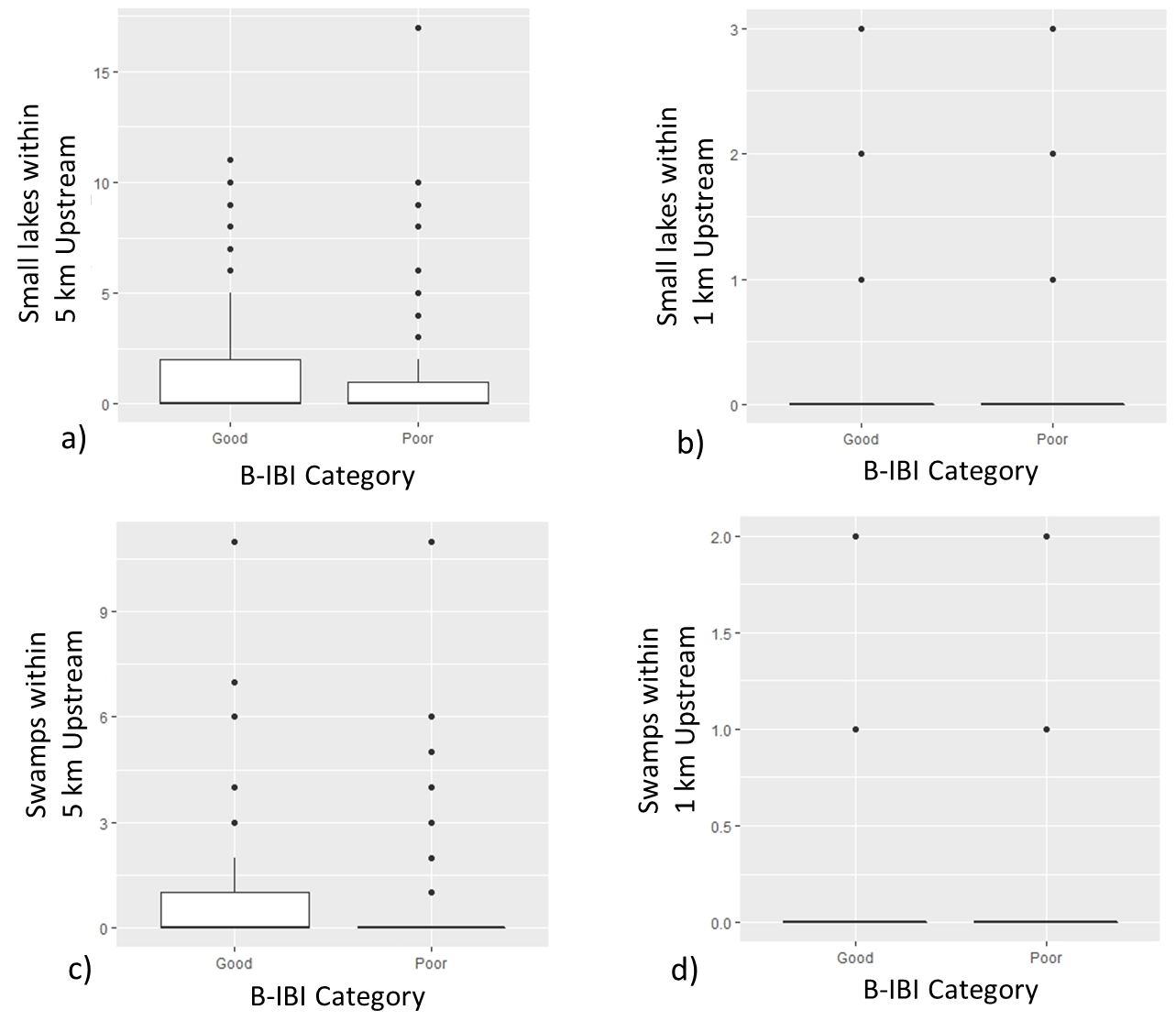
**

**
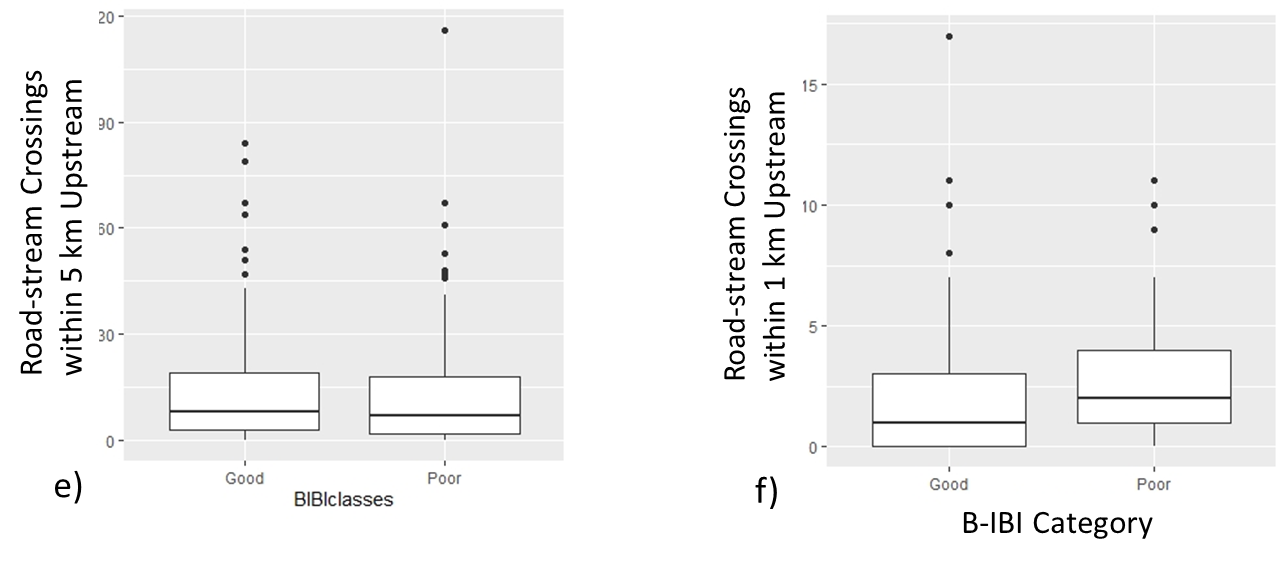
**

**
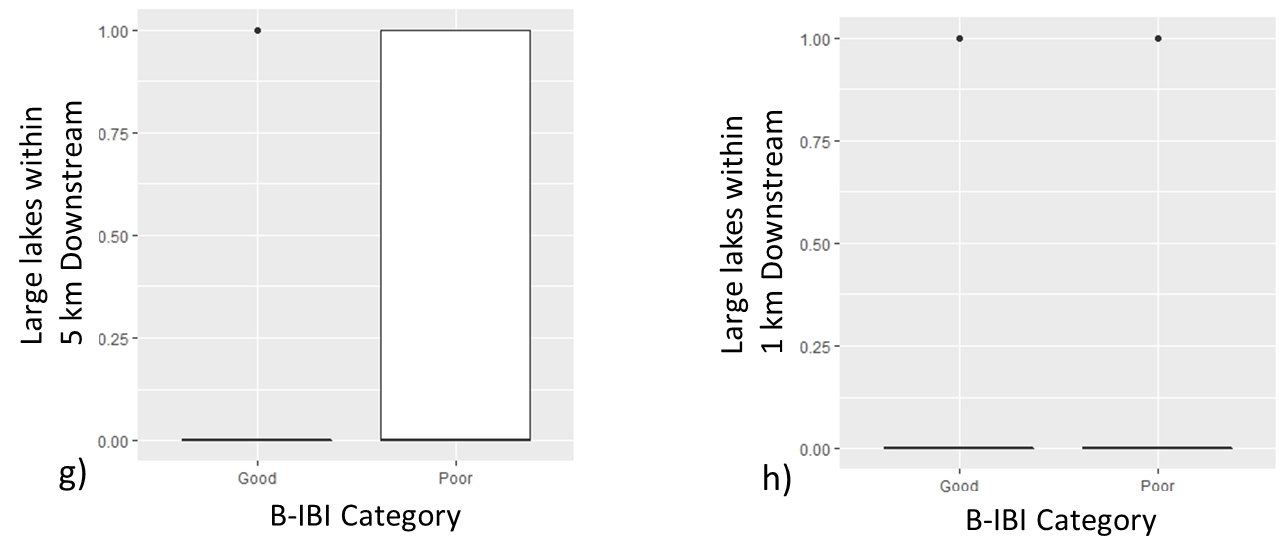
**

**
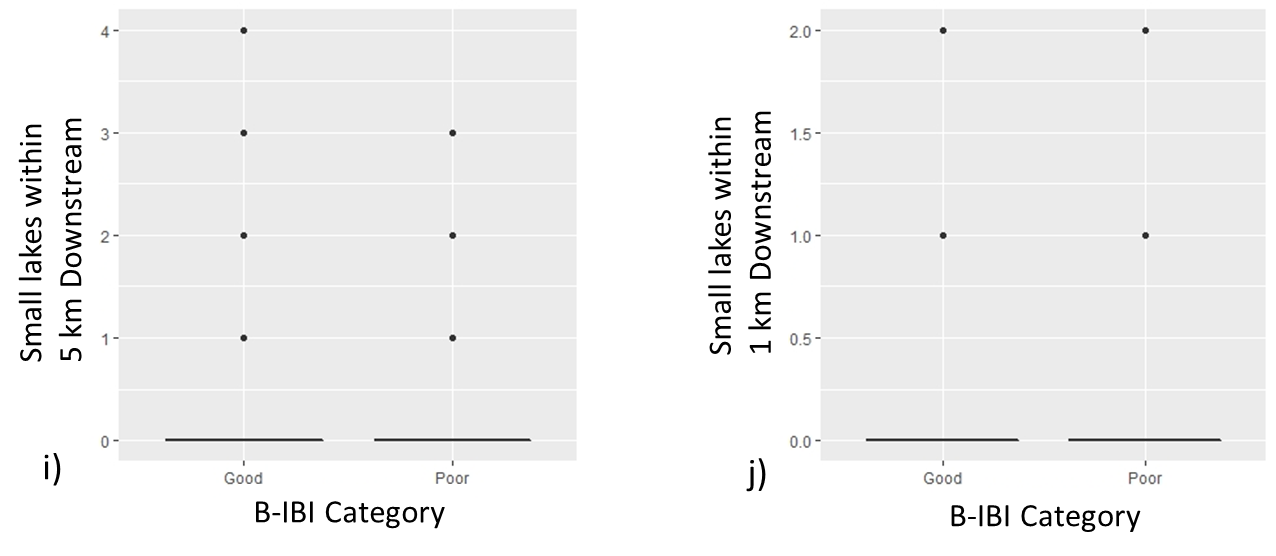
**

**
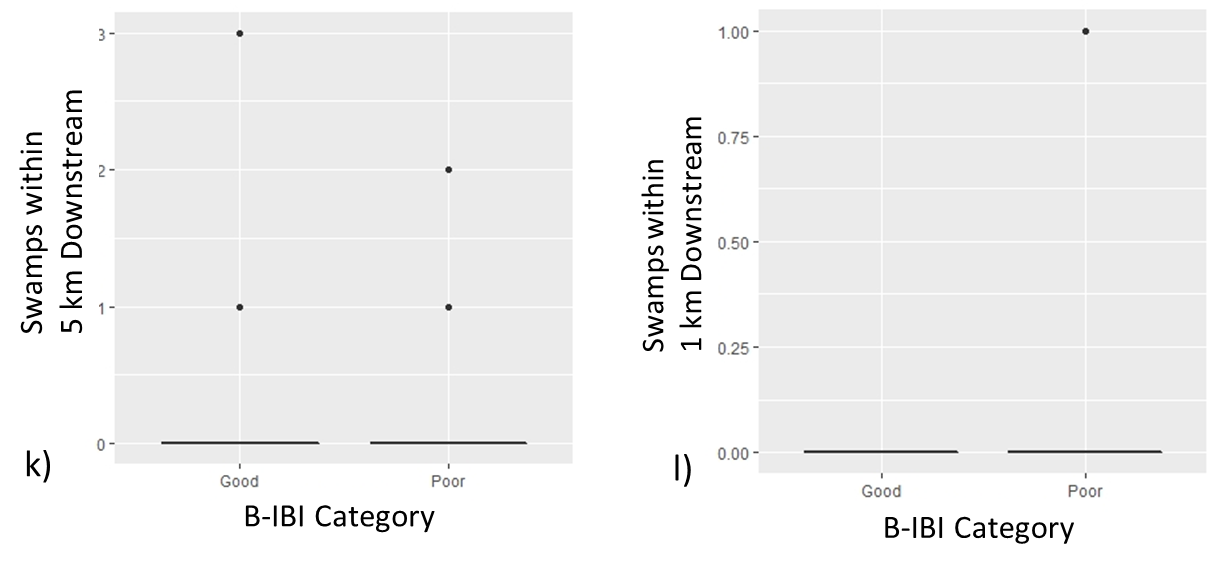
**

**
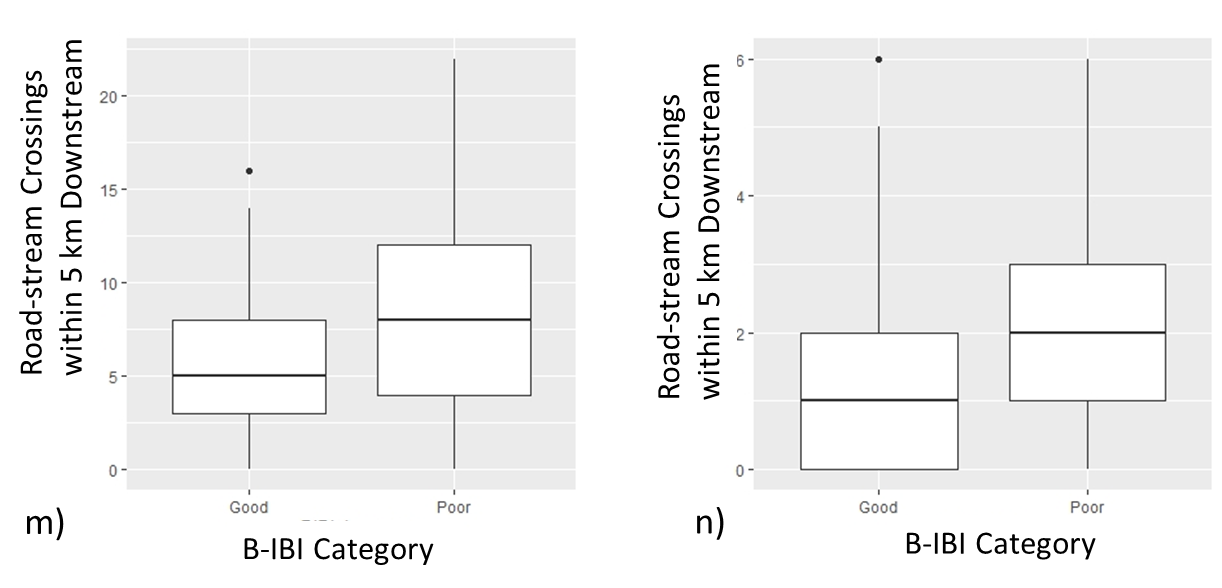
**

**
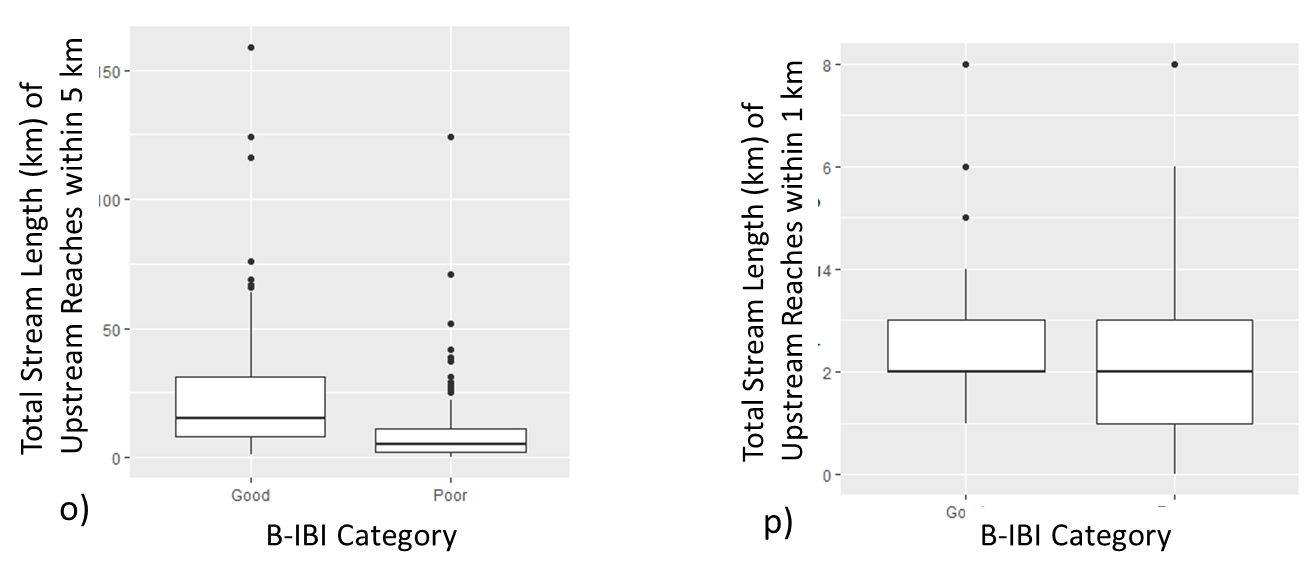
**

**
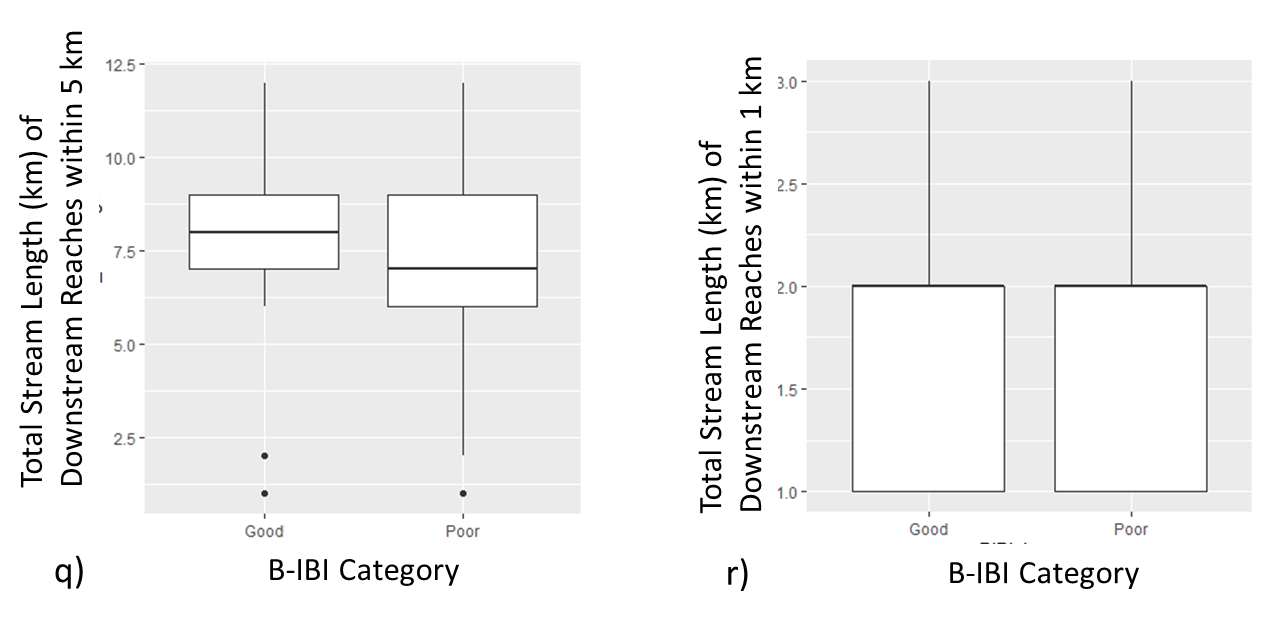
**
