# Supplementary material for: A multiscale landscape approach for prioritizing river and stream protection and restoration actions
Source: Ecosphere. Author manuscript; Available in PMC 2024 Jan 19. (PMC9903358; doi:10.1002/ecs2.4350)
Supplement: Supplement6 [file NIHMS1868745-supplement-Supplement6.docx]

**A multiscale landscape approach for prioritizing river and stream protection and restoration actions**

***Ecosphere***

Luisa Riato^1^, Scott G. Leibowitz^2^, Marc H. Weber^2^, Ryan A. Hill^2^

1. Oak Ridge Institute for Science and Education (ORISE) Post-Doctoral Fellow c/o U.S. Environmental Protection Agency, Center for Public Health and Environmental Assessment, Pacific Ecological Systems Division, 200 SW 35^th^ St., Corvallis, OR 97333 USA; [riato.luisa@epa.gov](mailto:riato.luisa@epa.gov)
2. U.S. Environmental Protection Agency, Center for Public Health and Environmental Assessment, Pacific Ecological Systems Division, 200 SW 35^th^ St., Corvallis, OR 97333 USA; leibowitz.scott@epa.gov, weber.marc@epa.gov, hill.ryan@epa.gov

**Appendix S1. Description of using network navigation to select upstream and downstream segments within a 5 km buffer range of each site**

We developed code in R statistical software (R Development Core Team 2017), leveraging the *nhdplusTools* package (Blodgett 2018), as well as the *sf* package (Pebesma 2018), to derive upstream segments and downstream mainstem segments that did not extend beyond a 5km buffer of each site. Network navigation was performed first using functions in the *nhdplusTools* package which makes use of network topology (i.e., from – to information) in the NHDPlusHR dataset. We used network navigation first to ensure we only included flow-connected stream reaches for a site. We then clipped reaches gathered using flow navigation, upstream or downstream, using a 5km buffer. Since the 5 km distance was determined based on a circle centered around the sample site, this led to stream distances often greater than the 5 km Euclidean distance due to the meandering nature of the stream as well as the multiple branches that could exist upstream of a site.

**References**

Blodgett, D. 2018. nhdplusTools: Tools for Accessing and Working with the NHDPlus. US Geological Survey, Reston, VA (Version R package version 0.3.08) [https://code.usgs.gov/water/nhdplusTools].

Pebesma, E. 2018. Simple Features for R: Standardized Support for Spatial Vector Data. The R Journal 10:439–446.

R Development Core Team. 2017. R: A language and environment for statistical computing. R Foundation for Statistical Computing. Vienna, Austria.
